# Human monoclonal antibodies targeting **α**-Gal restrict IgE engagement of **α**-Gal syndrome allergens

**DOI:** 10.1172/JCI192370

**Published:** 2026-09-01

**Authors:** Hyeseon Cho, Youngsil Seo, Haewon Sohn, Shailesh K. Choudhary, Jeff Skinner, Ming Zhao, Ludmila Krymskaya, Weizhi Zhong, Justin Lack, Shanping Li, Boubacar Traore, Joshua Tan, Scott P. Commins, Peter D. Crompton

**Affiliations:** 1 Malaria Infection Biology and Immunity Section, Laboratory of Immunogenetics, Division of Intramural Research, National Institute of Allergy and Infectious Diseases, NIH, Rockville, Maryland, USA.; 2 Division of Allergy and Immunology, University of North Carolina at Chapel Hill, Chapel Hill, North Carolina, USA.; 3 Protein and Chemistry Section, Research Technologies Branch, National Institute of Allergy and Infectious Diseases,; 4 Laboratory of Immunogenetics, Division of Intramural Research, National Institute of Allergy and Infectious Diseases,; 5 Antibody Biology Unit, Laboratory of Immunogenetics, Division of Intramural Research, National Institute of Allergy and Infectious Diseases, NIH, Rockville, Maryland, USA.; 6 Integrated Data Sciences Section, Research Technologies Branch, National Institute of Allergy and Infectious Diseases, NIH, Bethesda, Maryland, USA.; 7 Mali International Center of Excellence in Research; Malaria Research and Training Center, University of Sciences, Techniques and Technologies of Bamako, Bamako, Mali.

**Keywords:** Immunology, Infectious disease, Allergy

## Abstract

Allergen-specific monoclonal antibodies (mAbs) that block IgE binding to allergens are emerging as new therapeutics for treating allergies to pollen, peanuts, and cats. Alpha-Gal syndrome (AGS) is an allergy to galactose-α-1,3-Galactose (α-Gal), which is present in mammalian meat and tissue-derived products. Initially aiming to identify mAbs targeting α-Gal on malaria parasites, we isolated 42 α-Gal–specific mAbs from B cells of individuals who had been exposed to malaria but found that they bound weakly to the *Plasmodium falciparum* parasite. These mAbs predominantly used the *IGHV3* gene family and had a wide range of mutation frequencies. We then screened these mAbs for their ability to bind α-Gal on AGS allergens and to block the binding of serum IgE of patients with AGS to AGS allergens. Thirteen mAbs bound to the AGS allergens angiotensin-I-converting enzyme (ACE), aminopeptidase-N (AP-N), and cetuximab, and 2 mAbs— AG028 as both IgA_2_ and IgM, and AG050 IgA_1_ — blocked the binding of serum IgE from patients with AGS to ACE and AP-N. Additionally, AG028 IgA_2_ and AG028 IgM suppressed ACE-mediated activation of basophils sensitized with serum of patients with AGS. This study supports the development of α-Gal–specific mAbs as a new intervention to prevent α-Gal allergy.

## Abstract 2

This study demonstrates the proof of concept that alpha-Gal-targeting monoclonal antibodies could prevent alpha-Gal allergy.

## Graphical abstract

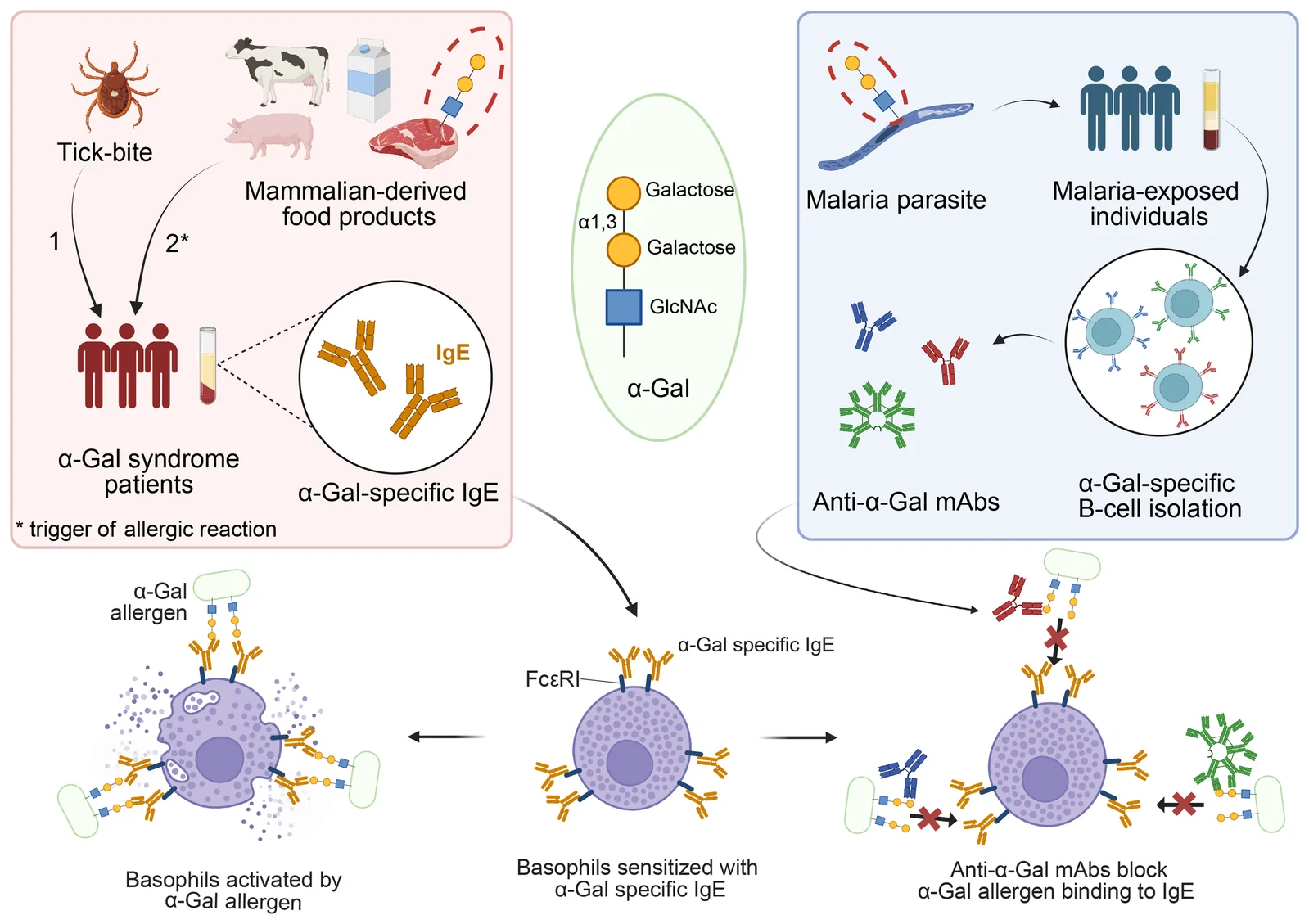

## Introduction

Alpha-Gal syndrome (AGS) is an emerging tick bite–associated allergy characterized by potentially life-threatening IgE-mediated reactions to galactose-α-1,3-Galactose (α-Gal) in red meat or other products containing α-Gal (1). Approximately 110,000 suspected cases of AGS, likely a substantial underestimate, have been identified since 2010 in the United States with no treatment or cure currently available (2). Individuals who suffer from AGS can experience a wide array of symptoms of variable severity, ranging from urticaria and gastrointestinal problems to anaphylaxis, typically 2–6 hours after exposure to α-Gal–containing products (3). On the other hand, the first exposure to intravenous cetuximab, a chimeric human/mouse IgG_1_ monoclonal antibody (mAb) approved for the treatment of colorectal cancer, can elicit immediate-onset anaphylaxis due to the presence of α-Gal on its mouse fragment antigen-binding (Fab) portion (4, 5). The most likely source of primary sensitization to α-Gal is tick bites that induce α-Gal–specific IgE (6–9). In the United States, the development of AGS is primarily associated with lone star tick bites (3). The association of AGS with tick bites has also been documented in Australia, Europe, and Japan, where lone star ticks are not found, suggesting that tick bites from various species may lead to the development of IgE antibodies to α-Gal (10–12).

The α-Gal epitope is a terminal sugar moiety present on mammalian glycoproteins and glycolipids, but it is not expressed in humans, apes, and old-world monkeys due to loss-of-function mutations in the gene encoding α1,3-Galactosyltransferase (GT) (13, 14). Loss of α-Gal expression resulted in the emergence of α-Gal–specific immunity, likely conferring resistance to α-Gal–expressing pathogens (15). Humans naturally produce IgM, IgA, and IgG antibodies (Abs) against the α-Gal epitope, and these anti-α-Gal Abs are probably sustained by α-Gal–expressing gastrointestinal flora (16–18). In patients with AGS, the mechanistic aspects of α-Gal IgE development have not been clearly elucidated. It has been proposed that tick bites initiate IgE class switching, probably in the skin, due to tick saliva–associated factors acting as IgE class switching adjuvants (19–21). Tick extract (TE) induced the proliferation of T and B cells with a Th2 cytokine profile more in patients with AGS than in controls. Additionally, a large proportion of TE-binding IgE and IgG_1_ Abs in patients with AGS were directed against the α-Gal epitope, and removing α-Gal from the TE reduced B cell proliferation in patients with AGS (22). Subcutaneous sensitization with TE or TE plus α-Gal in knockout mice lacking α1,3-GT, induces α-Gal-specific IgE, again supporting the view that sensitization occurs via the skin (23, 24). A recent study has reported that α-Gal IgE seropositivity alone does not predict symptomatic α-Gal allergy, implying the contribution of other host factors (25).

Allergen-induced cross linking of IgE bound to IgE receptors on effector cells such as basophils, eosinophils, and mast cells is the central event in acute IgE-mediated allergic reactions (26). Allergen-specific IgG or IgA, which interferes with IgE binding to allergens and thus prevents the cross-linking of IgE receptors, is likely an essential component of the protective mechanism of allergen immunotherapy (AIT) by inducing desensitization to allergens (27–29). Recent studies have demonstrated that passive administration of allergen-specific IgG mAbs provides symptom relief in individuals with birch and cat allergies and prevents peanut-induced anaphylaxis in mice (30–34).

In this study, we initially sought to identify human mAbs that target α-Gal on *Plasmodium falciparum* sporozoites — the first stage of malaria infection in humans — as a potential strategy to prevent malaria infection (15); as clinical trials in Africa have shown that mAbs targeting the *P*. *falciparum* circumsporozoite protein (*Pf*CSP) on sporozoites are protective against *P*. *falciparum* infection (35–37). However, the 42 α-Gal–specific mAbs we isolated from B cells of malaria-exposed individuals bound weakly to sporozoites. We then screened these 42 mAbs for their ability to bind α-Gal on AGS allergens and to block the binding of serum IgE of patients with AGS to AGS allergens. We found that 13 of these mAbs bound to the potent AGS allergens angiotensin-I-converting enzyme (ACE), aminopeptidase-N (AP-N), and cetuximab. Among these 13 mAbs, 2 blocked IgE in the serum of patients with AGS from binding to ACE and AP-N, and one interfered with ACE-mediated activation of basophils sensitized with AGS serum. In summary, anti-α-Gal mAbs could offer a new and targeted approach to preventing AGS by restricting the engagement of IgE with α-Gal allergens.

## Results

### Isolation of anti-α-Gal mAbs from malaria-exposed individuals.

In a study initially designed to identify mAbs that target α-Gal on *P*. *falciparum* sporozoites (15), we analyzed 1,053 plasma samples from a cohort study in Mali (Supplemental Figure 1; supplemental material available online with this article; https://doi.org/10.1172/JCI192370DS1) where intense malaria transmission occurs during the 6-month rainy season from July to December (38). Using a multiplex bead assay, we determined plasma levels of polyclonal α-Gal–specific IgG, IgM, and IgA in May at the end of the 6-month dry season (*n* = 550 participants) and in October at the peak of malaria transmission (*n* = 503 participants). α-Gal-specific IgM was the most prevalent isotype and was detectable in nearly all participants, in contrast to α-Gal–specific IgA and IgG, which were not detectable in approximately 10% and 30% of individuals, respectively. In a paired analysis, the median value of all individual median fluorescence intensities (median MFI) of IgA was approximately 2.8-fold higher in October than in May while the median MFI of IgG was approximately 1.7-fold higher (Supplemental Figure 1A). IgM also trended higher in October (Supplemental Figure 1A). Increases in IgA and IgG from May to October were the most pronounced in infants with 3.7- and 493-fold increases, respectively, who generally had low α-Gal-specific IgA and IgG responses in May at the end of the dry season (Supplemental Figure 1B). Ab responses specific to α-Gal generally showed a positive correlation with age, except for a statistically significant decrease in IgM median MFI from the middle to oldest age group at the May time point (Supplemental Figure 1B).

Next, we isolated α-Gal–specific mAbs from B cells of the 18 participants with the highest polyclonal antibody responses to α-Gal (Supplemental Table 1). Using synthetic α-Gal conjugated to HSA as a probe, 17,388 B cells were sorted from PBMCs of these 18 participants (Supplemental Figure 2) and distributed onto 384-well plates (average 1–2 cells per well). After 2 weeks of B cell activation, which allowed memory B cells to proliferate and differentiate into antibody-producing cells, the supernatants were screened for secretion of α-Gal–binding Abs using a multiplex bead assay. We identified 85 α-Gal–specific B cell clones from which RT-PCR was performed to amplify genes encoding the heavy and light immunoglobulin chains. The resulting PCR products were sequenced, and, based on the sequence analysis using the International Immunogenetics Information System (IMGT) database (39), a panel of 42 fully human, α-Gal–specific recombinant mAbs were generated. All IgG and IgM clones were initially expressed as human IgG_1_ recombinant mAbs, while IgA clones were expressed as IgA_1_ or IgA_2_ monomers without the J chain. Binding of these mAbs to synthetic α-Gal beads was confirmed (Supplemental Figure 3A). Subsequently, 7 IgM clones (AG030, AG032, AG062, AG068, AG069, AG081, and AG082) in addition to one IgG (AG008) and 2 IgA (AG027 and AG028) clones were reexpressed as IgM with J chain, based on their binding activities to various α-Gal–expressing pathogens (data not shown). All 10 IgM mAbs exhibited enhanced binding to α-Gal beads compared with their IgG and IgA counterparts, likely due to increased avidity (Supplemental Figure 3B).

### The gene usage and mutation frequency of human memory B cell–derived α-Gal–specific mAbs.

**Figure 1:**
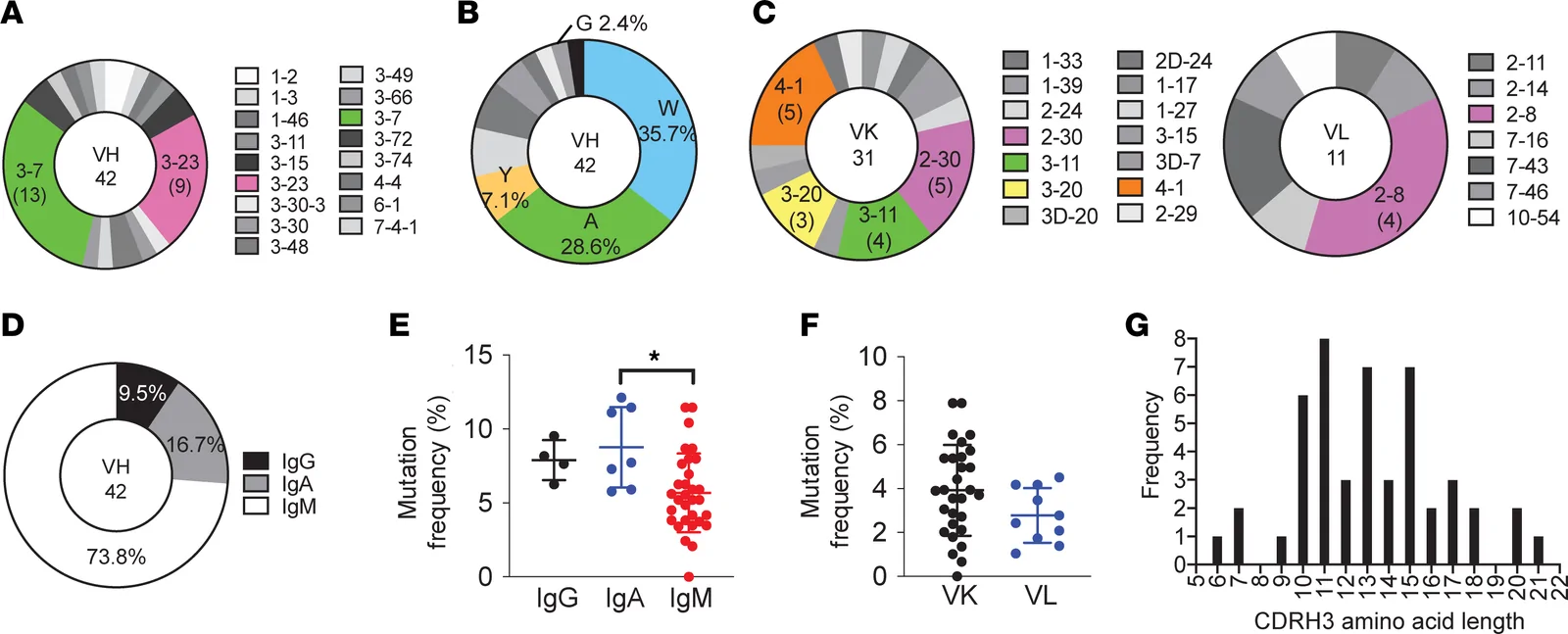
Gene usage, isotype distribution, and mutation frequencies among the 42 human anti-α-Gal mAbs. (**A**) VH gene usage. The 2 predominant genes, *IGVH3-7* and *IGVH3-23*, are highlighted with nonneutral colors. (**B**) Amino acid frequency at Kabat position 33 of CDRH1. (**C**) VK and VL gene usage. Frequently observed VK and VL genes are highlighted with nonneutral colors. (**D**) Isotype distribution of antibody heavy chains. (**E**) Mutation frequencies of VH genes in IgG, IgA, and IgM isotypes. (**F**) Mutation frequencies of VK and VL light chain genes. Mutation percentages were compared using Kruskal-Wallis and Dunn’s post hoc tests. *P < 0.05 (**G**) The CDR3 amino acid length of anti-α-Gal heavy chains. Numbers in parentheses indicate the exact number of clones.

The 42 memory B cell–derived mAbs specific for α-Gal utilized various VH and VL gene families, with a predominant use of the *IGHV3* gene family for VH, consistent with a prior report (Figure 1A) (40). Also of note, a previous study found that anti-α-Gal B cells preferentially use the *IGHV3-7* gene, particularly within the memory compartment (41). This gene encodes tryptophan at Kabat position 33 in the heavy chain complementarity-determining region 1 (CDRH1), which is crucial for the recognition of α-Gal (41). In the present study, we found that *IGHV3-7* was the most frequently observed VH gene, with a prevalence of 31% (13 clones) (Figure 1A); and, moreover, 12 of the 13 *IGHV3-7* clones encoded tryptophan at Kabat position 33 in CDRH1 (Supplemental Table 2). Overall, among all 42 mAbs, tryptophan and alanine were the most common residues at Kabat position 33 in CDRH1, with frequencies of 35.7% and 28.6%, respectively, while only one clone contained glycine, in contrast with a previous study that identified glycine as one of the 4 most common amino acids (Figure 1B) (41).

Approximately 74% of the 42 mAbs utilized the κ light chain, with no predominant gene usage observed for *IGKV* or *IGLV* (Figure 1C). Among the 42 mAbs, 31 were isolated as IgM (73.8%), 7 as IgA (16.7%), and 4 as IgG (9.5%) (Figure 1D and Supplemental Table 2), which is consistent with a previous study showing that α-Gal–specific Abs are predominantly IgM (41). The α-Gal–specific mAbs exhibited a wide range of mutation frequencies, with heavy and light chain gene mutation frequencies ranging from 0%–12.1% and 0%–7.9%, respectively (Figure 1, E and F). The mutation frequencies of IgM VH varied the most, from 0%–11.5%, with a mean mutation frequency of 5.7%. The amino acid length of CDRH3 ranged from 6–21 with a mean of 14.8 (Figure 1G).

### α-Gal–specific mAbs do not bind strongly to Plasmodium sporozoites.

We screened the panel of 42 α-Gal–specific mAbs and ten mAbs reexpressed as IgM with J chain, for their ability to bind to live *Plasmodium* sporozoites. Unexpectedly, all mAbs exhibited minimal to very modest binding to the sporozoites, with the exception of AG062 IgM, which showed strong binding (Supplemental Figure 4, A–C). *Pf*CSP is the most abundant and immunodominant protein on the sporozoite surface (42). Previous research by Yilmaz et al. investigated whether *Pf*CSP had an α-Gal modification, but their findings were inconclusive (15). To determine if AG062 IgM could bind to *Pf*CSP, we performed a multiplex bead assay using recombinant *Pf*CSP and chemically synthesized peptides containing various regions of *Pf*CSP, including the immunodominant NANP repeat, as described in a prior study (43). Surprisingly, AG062 IgM bound to the *Pf*CSP NANP repeat, which does not contain α-Gal. This suggests that the binding of AG062 IgM to sporozoites is likely due to cross-reactivity with the NANP repeat of *Pf*CSP (Supplemental Figure 4D). Yilmaz et al. also demonstrated that sera from α1,3-GT and activation-induced cytidine deaminase–deficient mice immunized with rabbit red blood cell membranes (polyclonal IgM), as well as an α-Gal–specific mouse mAb in IgG2b and IgG3 isotypes, bound to *Plasmodium* sporozoites and conferred protection against *Plasmodium* infection in α1,3-GT-deficient mice (15). This suggests the existence of multiple configurations of the α-Gal epitope, and that the α-Gal–specific mAbs isolated in this study have low-affinity binding to the form of α-Gal on *P*. *falciparum* sporozoites. Therefore, we explored whether these mAbs could bind to AGS allergens.

### Human α-Gal–specific mAbs isolated from malaria-exposed individuals bind AGS allergens.

**Figure 2:**
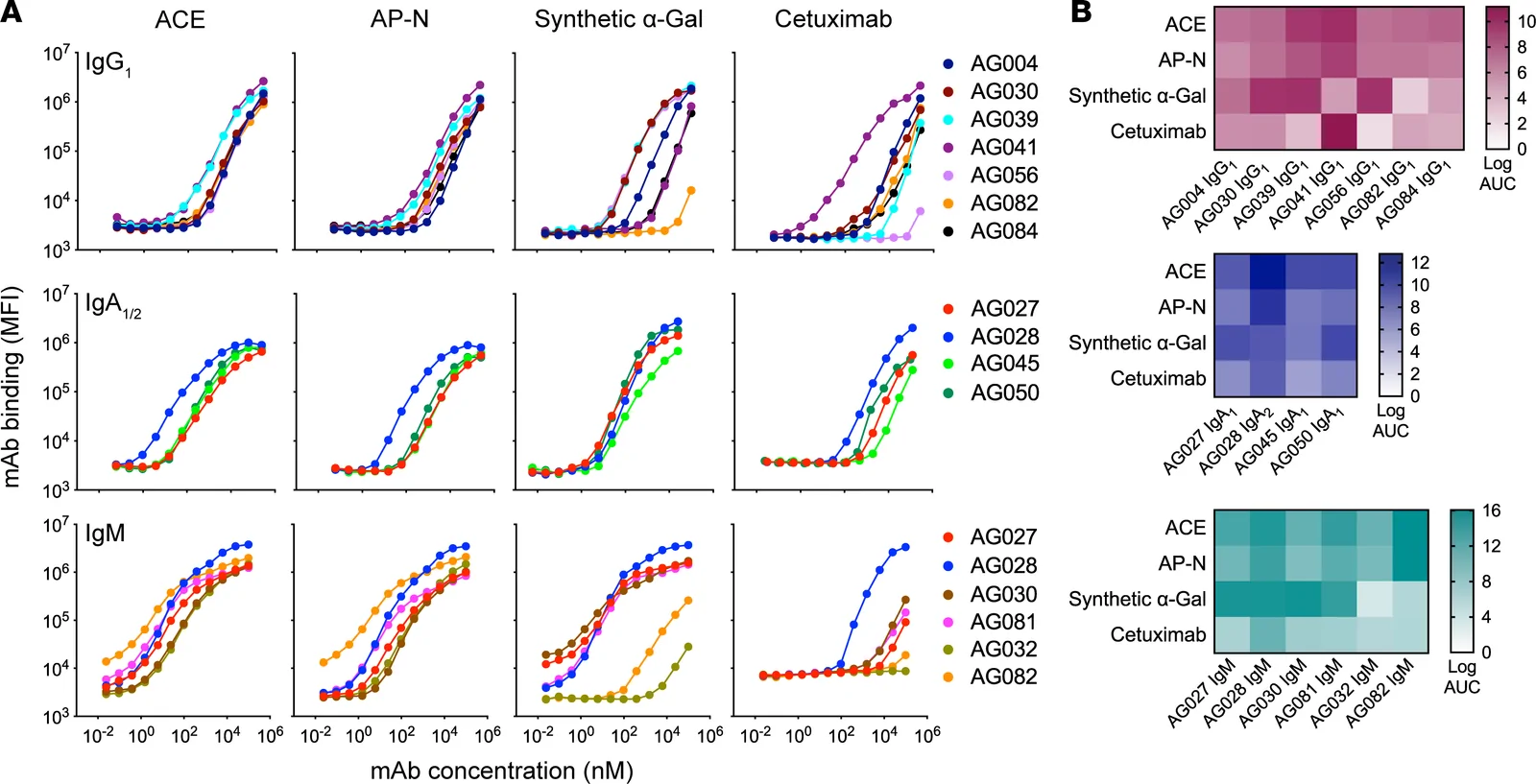
Binding of anti-α-Gal mAbs to ACE, AP-N, synthetic α-Gal–conjugated to BSA, and cetuximab. Titration curves (**A**) and AUC heat maps (**B**) of 17 anti-α-Gal mAbs (derived from 13 anti-α-Gal mAb clones) binding to the AGS allergens ACE, AP-N, synthetic α-Gal, and cetuximab. For the heat maps, the color scale ranges from dark [highest log_10_(AUC) values in each isotype] to white [log_10_(AUC) = 0]. For IgG_1_ mAbs binding to cetuximab, the F(ab′)_2_ fragment of cetuximab was used.

We evaluated whether the anti-α-Gal mAbs, which showed higher binding to synthetic α-Gal conjugated to BSA, could bind other AGS allergens. In addition to synthetic α-Gal conjugated to BSA, 3 naturally derived AGS allergens were included. ACE and AP-N, purified from porcine kidney, have been identified as major IgE-reactive α-Gal–containing glycoproteins and are known as potent triggers of red meat–induced delayed anaphylaxis in patients with AGS. Cetuximab, a therapeutic IgG_1_ chimeric antibody consisting of human constant regions and mouse variable regions, is used in colorectal cancer treatment. Since cetuximab is expressed in the murine SP2/0 cell line, it can similarly induce severe hypersensitivity reactions through α-Gal epitopes present on the Fab portion of its heavy chain (4, 5). The concentration-dependent binding of anti-α-Gal mAbs to ACE, AP-N, and cetuximab was evaluated using allergen binding titration curves (Figure 2A). The overall extent of this binding was then quantified and visualized with AUC heat maps (Figure 2B). To avoid false positives from the secondary antibody recognizing the human IgG_1_ Fc region of cetuximab, we digested cetuximab with pepsin and purified the resulting F(ab′)_2_ fragments for use in the binding assays.

Analysis of the panel, comprising 13 mAbs and 4 reexpressed IgM mAbs, revealed 3 distinct binding patterns across the tested allergens. First, AG030 (IgG1 and IgM isotypes) and AG056 (IgG1) had higher activity against synthetic α-Gal than natural allergen sources, suggesting a preference for structural conformations that are more abundant in synthetic constructs. Second, AG041 (IgG1), AG082 (IgG1 and IgM isotypes), and AG032 (IgM) had higher affinity for natural allergen sources over synthetic α-Gal, suggesting recognition of extended epitopes or conformational features unique to native glycoproteins. Third, AG028 (IgA2 and IgM isotypes) and AG050 (IgA_1_) demonstrated broad cross reactivity, with consistently high binding activity across all allergens tested. This pattern suggests recognition of a core α-Gal structural determinant that is conserved regardless of its presentation context. Collectively, these results not only confirm the binding of these mAbs to AGS-relevant allergens but also reveal heterogeneity in their recognition patterns, which is dependent on the specific mAb and the nature of the allergen source.

### IgE and IgG_1–4_ responses of patients with AGS to known AGS allergens.

Next, with the aim of evaluating the potential functional relevance of the 13 α-Gal–specific mAbs and 4 reexpressed IgM mAbs that bound to AGS allergens, we characterized the serologic profiles of serum samples collected from a cohort of patients with AGS. In total, 88 serum samples from patients with AGS at the University of North Carolina (UNC), Chapel Hill allergy clinic, and 31 plasma samples from healthy adult controls at the UNC clinic and National Institutes of Health, were analyzed. Detailed characteristics of the patients with AGS, including previously determined total and α-Gal–specific IgE levels, are provided in Supplemental Table 3. Serum of patients with AGS had α-Gal–specific IgE levels ranging from 0.1 to > 100 international units (IU)/mL (Supplemental Table 3).

We first profiled the serum samples of this cohort using a multiplex bead assay to determine polyclonal antibody levels to ACE, AP-N, and cetuximab. The multiplex assay also included the α-Gal–containing milk allergens lactoferrin (LF), lactoperoxidase (LPO), and bovine γ-globulin (BGG) (44), which are considered weak AGS inducers, given that approximately 80%–90% of patients with AGS do not react to milk or milk products (45). We also examined antibody responses to bovine and porcine gelatin, since it was unclear if gelatin could trigger an AGS-related reaction (46). Synthetic α-Gal (α-Gal trisaccharide linked to BSA) and human thyroglobulin, which is highly glycosylated but lacks α-Gal, were used as positive and negative controls, respectively (47).

**Figure 3:**
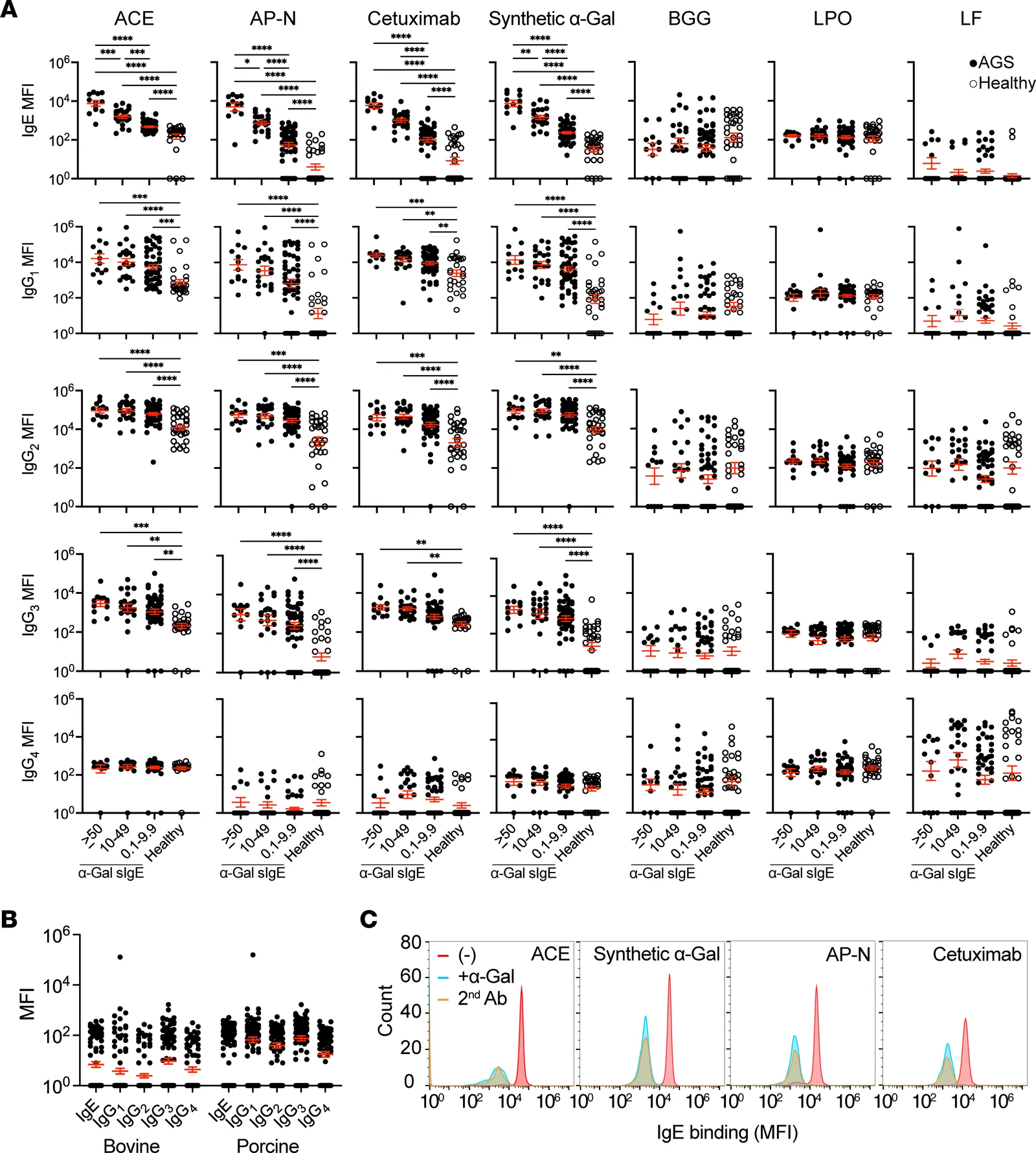
Antibody responses to AGS allergens in serum of patients with AGS. (**A**) Assessment of serum IgE and IgG_1–4_ responses of patients with AGS and healthy US individuals to ACE, AP-N, cetuximab, BGG, LF, LPO, and synthetic α-Gal–conjugated to BSA as determined by a multiplex beads assay. 88 AGS serum samples are divided into 3 groups based on the α-Gal–specific IgE levels: ≥ 50 IU (*n* = 12), 10–49 IU (*n* = 22), and 0.1–9.9 IU (*n* = 52). The serum samples of participant UNC0209 were used at 2 different time points due to different α-Gal–specific IgE levels. Healthy US individuals, *n*=31. For IgG_1_ reactivity, the F(ab′)_2_ of cetuximab was used. The MFI of human thyroglobulin (the negative control) was subtracted from each value. (**B**) AGS patient serum antibody responses to bovine and porcine gelatin. ANOVA multiple comparisons: *P < 0.05; **P < 0.01; ***P < 0.001; ****P < 0.0001. (**C**) IgE levels of UNC0383.1 serum to allergens in the absence (−) or presence (+α-Gal) of 10 mM α-Gal trisaccharide, and the secondary antibody–only control (2^nd^ Ab).

Compared to healthy controls, patients with AGS had elevated IgE reactivities to ACE, AP-N, and cetuximab, but not to milk proteins (Figure 3A and Supplemental Figure 5). Their IgE responses to ACE, AP-N, and cetuximab were similar to their IgE responses to synthetic α-Gal (Figure 3A).

Next, we assessed IgG_1–4_ responses to the AGS allergens, since prior studies reported that α-Gal–specific IgG levels were elevated in patients with AGS compared with healthy individuals and could be used as a potential marker of AGS risk (48, 49). As mentioned earlier, we used a cetuximab F(ab′)_2_ fragment to assess IgG_1_ mAbs. Patients with AGS showed higher IgG_1–3_, but not IgG_4_ binding to ACE, AP-N, synthetic α-Gal, and cetuximab compared with healthy individuals (Figure 3A). IgG_1–3_ levels to the milk allergens were similar between patients with AGS and healthy controls and tended to be lower than responses to ACE, AP-N, and cetuximab (Figure 3A). Among patients with AGS, we found little to no IgE and IgG_1–4_ responses to bovine and porcine gelatin except for 1 individual who had high IgG_1_ levels to bovine and porcine gelatin as well as milk allergens (Figure 3, A and B).

Next, we tested if soluble α-Gal could abolish serum IgE binding to the α-Gal–containing allergen beads to determine whether the IgE responses were specific to the α-Gal moieties. In the presence of 10 mM soluble Gal-α1,3-Gal-β1,4-GlcNAc trisaccharide (soluble α-Gal), the 4 AGS serum samples (UNC0146.2, UNC0147.1, UNC0209.1, and UNC0383.1) with the highest IgE levels to ACE, AP-N, cetuximab, and synthetic α-Gal lost IgE binding to these antigens, confirming that the IgE responses are mainly directed to α-Gal (Figure 3C and Supplemental Figure 5B).

**Figure 4:**
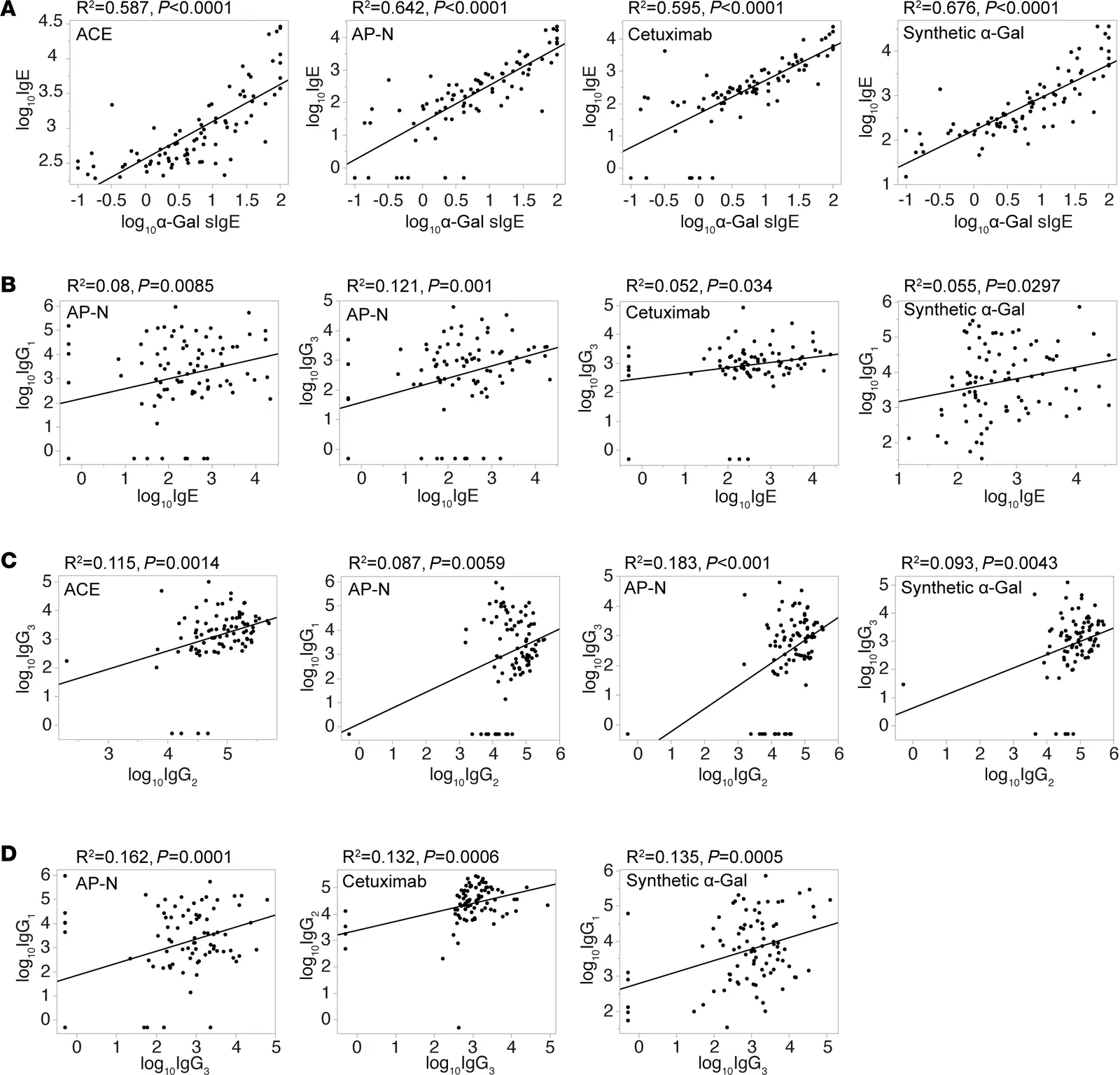
Correlation of reactivities to ACE, AP-N, synthetic α-Gal–conjugated to BSA, and cetuximab between IgE and IgG subclasses in serum from patients with AGS. Graphs were plotted MFIs of 88 patient serum antibodies reactive to the allergen-specific IgE and IgG subclass measured in Figure 3 and α-Gal–specific IgE levels in Supplemental Table 3 were analyzed for correlation. (**A**) Correlations between α-Gal–specific IgE levels and IgE MFIs specific to ACE, AP-N, cetuximab, and synthetic α-Gal. (**B**) Correlations between IgE MFIs and IgG subclass MFIs for the indicated antigens. (**C**) Correlations between IgG_2_ and IgG subclass MFIs for the indicated antigens. (**D**) Correlations between IgG_3_ and IgG subclass MFIs for the indicated antigens. Correlation coefficients (r) and *P* values were determined by linear fitting using JMP software. Only statistically significant correlations with r^2^ > 0.05 are shown.

As expected, we found a strong correlation between the MFI of IgE reactive to ACE, AP-N, and cetuximab measured by multiplex bead assay and the α-Gal–specific IgE levels (IU) shown in Supplemental Table 3 (Figure 4A). In contrast, bead-assay measurements of serum antibody reactivities to ACE, AP-N, and cetuximab showed no or only weak correlations between IgE and IgG_1–3_ as well as between the individual IgG subclasses (Figure 4, B–D). These findings suggested that ACE, AP-N, and cetuximab could be used as representative AGS allergens to further characterize the panel of α-Gal–specific mAbs described above.

### Anti-α-Gal mAbs block IgE binding to ACE and AP-N.

**Figure 5:**
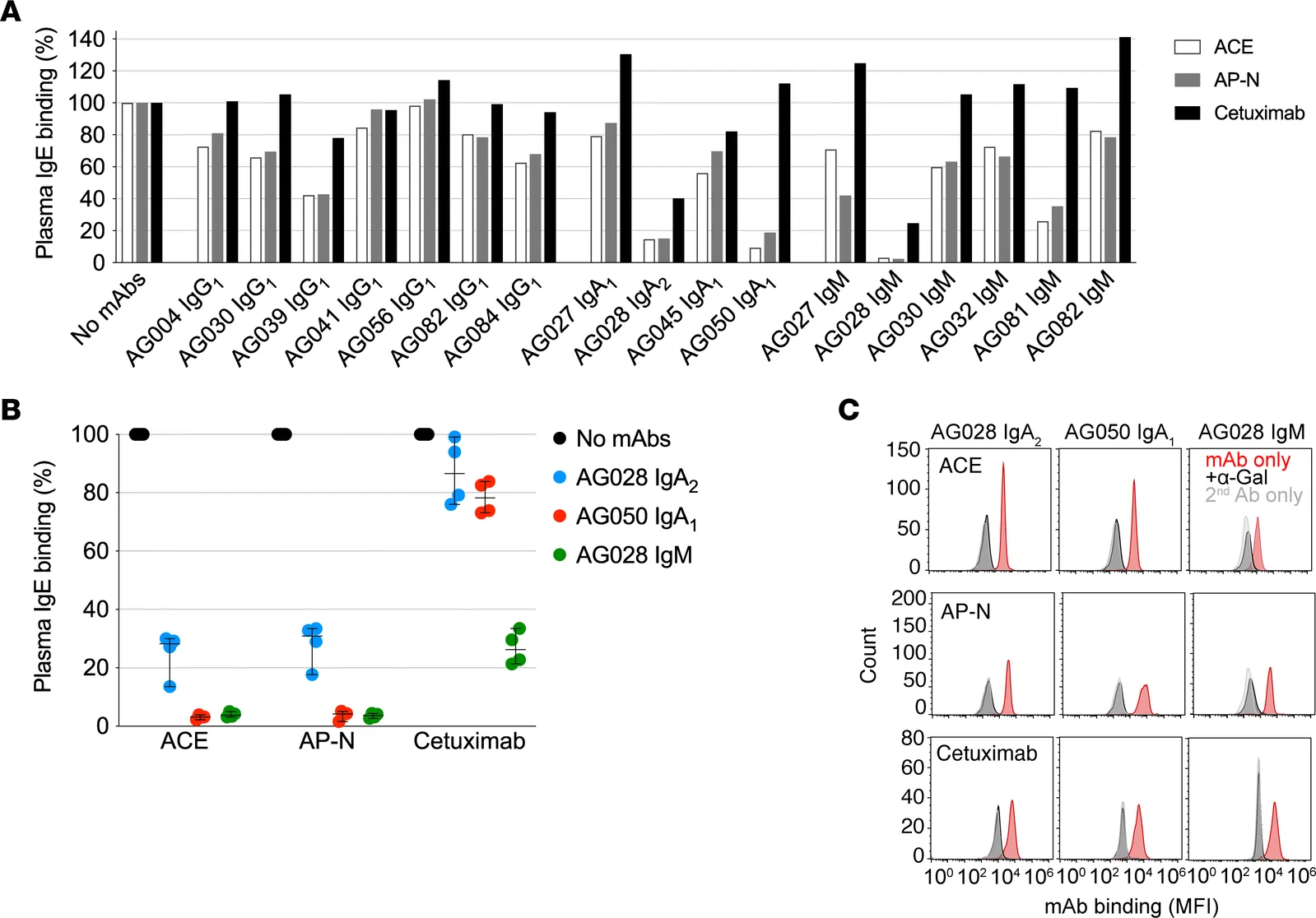
Anti-α-Gal mAbs interfere with polyclonal IgE binding of serum from patients with AGS to ACE, AP-N, and cetuximab. (**A**) Screening of anti-α-Gal mAbs generated in CHO cells for blocking UNC0209.1 AGS serum IgE binding to ACE, AP-N, and cetuximab. (**B**) Inhibition by AG028 IgA_2_, AG050 IgA_1_, and AG028 IgM mAbs produced in Expi293F cells (50 μg/mL) of polyclonal IgE binding of 4 diluted AGS serum samples (UNC0146.2, UNC0147.1, UNC0209.1, and UNC0383.1). Mean ± SD. (**C**) Soluble α-Gal competition with anti-α-Gal mAbs for binding to ACE, AP-N, and cetuximab. 10 mM of α-Gal trisaccharide was preincubated with mAbs before adding the allergen beads.

We used serum samples of patients with AGS to determine if the 13 α-Gal–specific mAbs and 4 reexpressed IgM mAbs that bound to AGS allergens could block IgE-binding to ACE, AP-N, and cetuximab. For the initial screen, due to limited availability of serum samples from patients with AGS, we only tested inhibition with the serum sample of patient UNC0209.1 (1 of the 4 serum samples that showed the highest IgE reactivity to AGS allergens). We preincubated the α-Gal–specific mAbs with the allergen-bound beads, added serum from AGS patient UNC0209.1, and then assessed the degree of inhibition of serum IgE-binding to AGS allergens (Figure 5A). The mAb AG028, both as an IgA_2_ and an IgM, interfered with IgE-binding to all 3 allergens (approximately 60%–97% inhibition), whereas AG050 IgA_1_ inhibited IgE-binding only to ACE and AP-N (approximately 81%–91% inhibition). The remaining mAbs showed low-to-modest inhibition (less than 60% inhibition).

The recombinant mAbs were initially generated in Chinese hamster ovary (CHO) cells which express α1,3-GT. Although it has been reported that anti-α-Gal IgE from patients with AGS is not reactive to therapeutic mAbs generated in CHO cells (50), we reexpressed AG028 IgA_2_, AG050 IgA_1_, and AG028 IgM in human Expi293F cells that lack α1,3-GT activity. Since different cell culture conditions can result in variability in mAb characteristics, potentially through posttranslational modification (51), we compared CHO cell– versus Expi293F cell–generated mAbs for allergen binding and interference with AGS serum IgE binding. mAbs expressed in Expi293F cells compared with those expressed in CHO cells exhibited similar binding to the allergens and comparable inhibition of AGS serum IgE binding to allergens (Supplemental Figure 6).

Next, we used 4 serum samples from patients with AGS (UNC0146.2, UNC0147.1, UNC0209.1, and UNC0383.1) with the highest IgE reactivities to AGS allergens to assess whether mAb AG028, as an IgA_2_ and an IgM, and AG050 IgA_1_ showed similar inhibition with different AGS serum samples. Across all 4 serum samples, all 3 mAbs inhibited AGS serum IgE binding to ACE and AP-N (Figure 5B). AG028 IgA_2_ and AG050 IgA_1_ showed minimal inhibition of IgE binding to cetuximab, compared with approximately 70% inhibition by AG028 IgM (Figure 5B). The 2 mAbs that most effectively inhibited IgE binding to ACE and AP-N were AG028 IgA_2_/IgM and AG050 IgA_1_. To confirm that AG028 IgA_2_, AG050 IgA_1_, and AG028 IgM bind to α-Gal on ACE and AP-N, we incubated these mAbs with soluble α-Gal, which resulted in a loss of binding to ACE, AP-N, and cetuximab (Figure 5C).

### Competition and binding affinity analysis of anti-α-Gal mAbs.

Next, we performed competition assays to determine whether the α-Gal–specific mAbs bind overlapping or distinct epitopes and consequently compete with one another for antigen binding. In addition to AG028 and AG050 IgAs, 2 additional α-Gal–specific antibodies were included: the mouse IgM M86 and AG056 IgG_1_, each with distinct binding properties. M86, originally isolated against rabbit red blood cell membranes, has been widely used to study the structural basis of α-Gal–specific Ab responses (41, 52). AG056 IgG_1_ exhibited minimal inhibition of serum IgE binding (Figures 5A).

**Figure 6:**
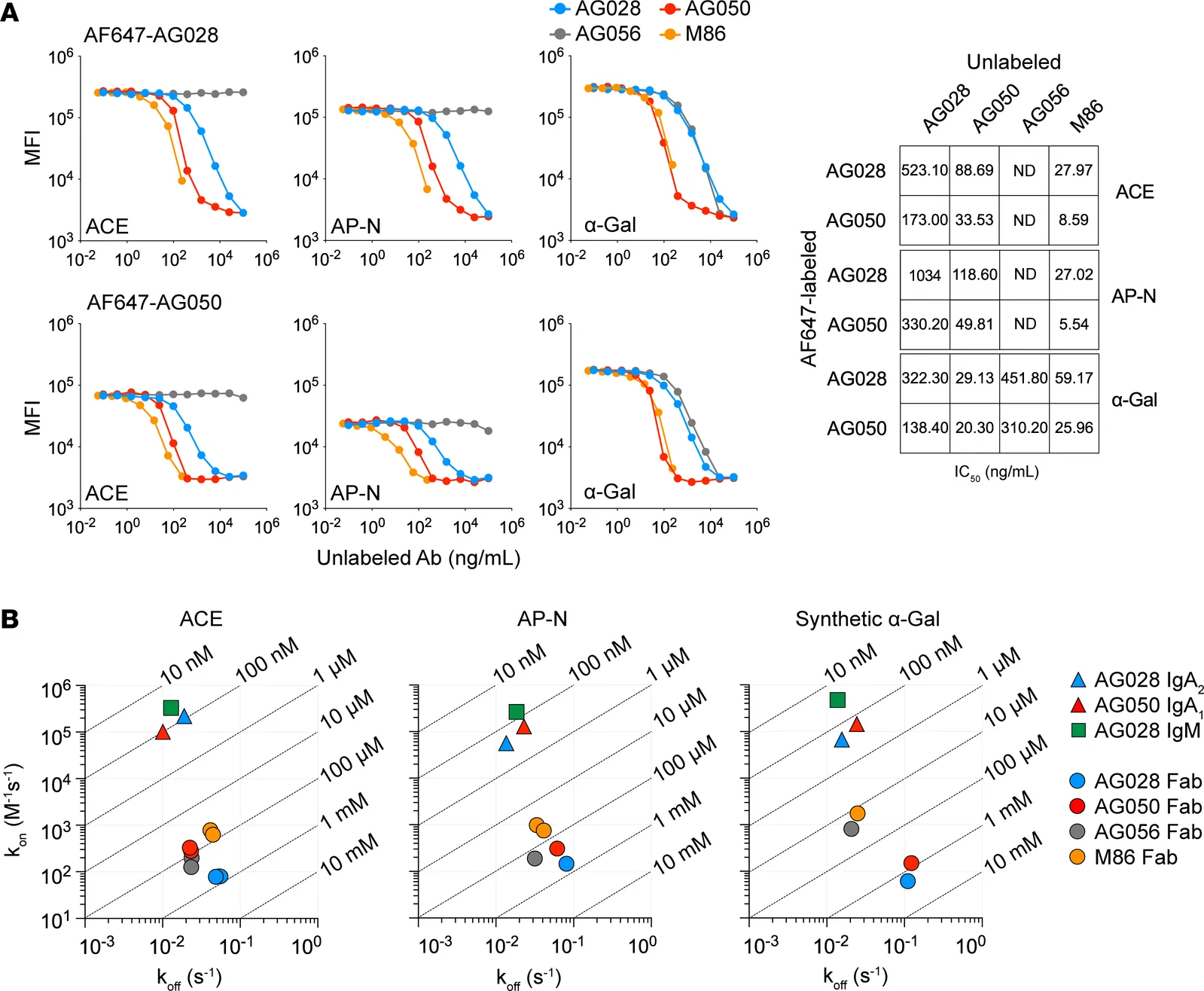
Competition and binding kinetics of AG028, AG050, AG056, and M86 antibodies to AGS allergens. (**A**) Anti-α-Gal mAbs binding competition assay to allergen-conjugated beads. AF647-AG028 IgA_2_ (1 μg/mL) or AF647-AG050 IgA_1_ (4 μg/mL) was competed for binding to allergen-conjugated beads with increasing concentrations (0.01 ng/mL to 100 μg/mL; 1:4 serial dilution) of the unlabeled human mAbs AG028 IgA_2_, AG050 IgA_1_, and AG056 IgG_1_, and the unlabeled mouse mAb M86 IgM. IC_50_ values were calculated using Prism software with a 4-parameter logistic (4PL) sigmoidal model (X = concentration); curves with R^2^ > 0.95 are shown; those with R^2^ < 0.95 were designated as not determined (ND). (**B**) Two-dimensional isoaffinity kinetic plots show the association (k_on_) and dissociation (k_off_) rate constants for antibody binding to ACE, AP-N, and synthetic α-Gal. Each allergen was immobilized on streptavidin biosensors, and binding kinetics were measured using the Octet system. Dashed diagonal lines indicate equilibrium dissociation constants (K_D_) to facilitate visualization of affinity distributions. The avidities of multivalent antibodies (AG028 IgA_2_, AG050 IgA_1_, and AG028 IgM) and affinities of corresponding monovalent Fab fragments (AG028, AG050, AG056, and M86) are shown. Binding kinetics of 4 Fabs for ACE and M86 Fab for AP-N were evaluated in 2 independent experiments, while the remaining samples were performed once.

Competition assays were performed in which Alexa Fluor 647–conjugated (AF647-conjugated) AG028 IgA2 or AF647–AG050 IgA1 was competed with unlabeled AG028 IgA2, AG050 IgA1, AG056 IgG1, or M86 IgM for binding to ACE, AP-N, or synthetic α-Gal, and IC_50_ values were determined for each unlabeled mAb (Figure 6A). Binding of both AF647–AG028 and AF647–AG050 to all α-Gal allergens was strongly competed by M86. In contrast, AG056 showed no competition for ACE or AP-N, but competition for synthetic α-Gal was comparable with AG028. Together, these findings indicate that AG028 and AG050 recognize overlapping α-Gal epitopes similar to M86, rather than epitopes with a preference for synthetic α-Gal. In direct competition between AG028 and AG050, IC_50_ values were consistently lower when AF647–AG028 was competed with unlabeled AG050 than when AF647–AG050 was competed with unlabeled AG028 across all allergens, indicating that AG050 may bind the allergens with higher apparent affinity than AG028 (Figure 6A).

To determine whether the observed serum IgE inhibition and competitive properties were driven by differences in binding affinity, we measured the binding affinities of 4 Abs in Fab format, as well as the apparent affinities of AG028 IgA_2_/M and AG050 IgA_1_ for ACE, AP-N, and synthetic α-Gal using biolayer interferometry (BLI) (Figure 6B, Supplemental Figures 7 and 8, and Supplemental Table 4). In contrast with IgA and IgM Abs, which exhibited strong apparent affinities due to their bi- and multivalent nature (K_D_ range: 28.95–239.4 nM), Fab fragments showed substantially weaker binding affinities, with K_D_ values ranging from 14.29 to 1,846 μM. Although the Fab affinities were generally too weak to allow precise quantitative comparisons, AG028 Fab consistently displayed the lowest binding affinity to all α-Gal allergens, despite AG028 exhibiting strong allergen binding in its IgA or IgM formats. Taken together, the BLI data indicate that differences in serum IgE inhibition and competitive behavior among anti-α-Gal mAbs cannot be explained solely by binding affinity.

### Anti-α-Gal mAbs suppress ACE-mediated activation of basophils sensitized with serum from patients with AGS.

**Figure 7:**
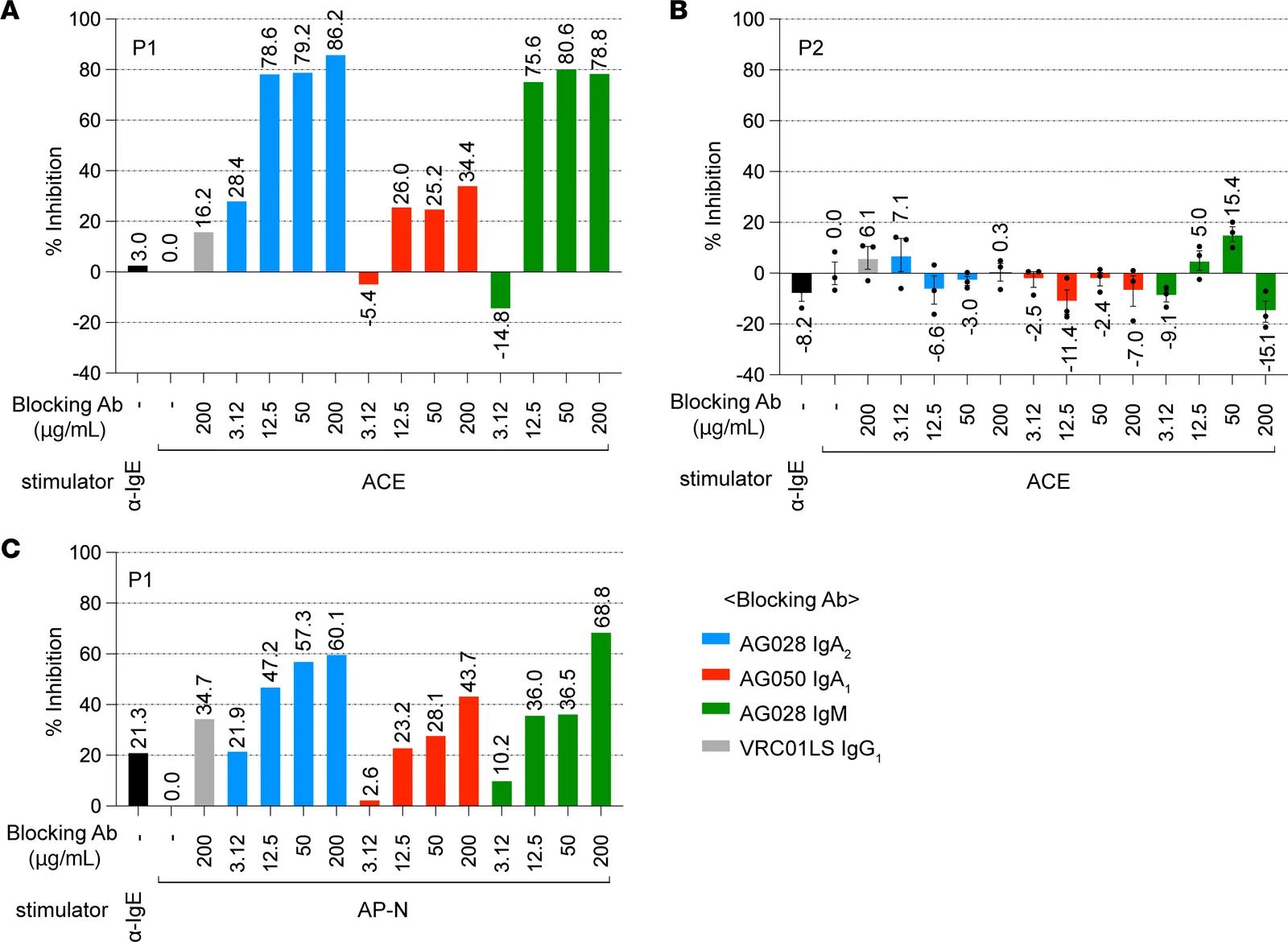
Indirect basophil activation test. Basophils within PBMCs from healthy individuals were stripped of IgE with cold lactic acid and sensitized overnight with serum samples of 2 patients with AGS (P1, UNC0445.1 and P2, UNC0512.1). The sensitized cells were stimulated with 2 ng/mL of IL-3 and 1 mg/ml of ACE or AP-N for 30 minutes at 37°C in the presence or absence of AG028 IgA_2_, AG050 IgA_1_, and AG028 IgM at concentrations of 3.12, 12.5, 50, and 200 mg/mL, and then the percentage of lineage-negative, HLA-DR negative, CD123/CD203c-positive basophils that were CD63^+^ was assessed by flow cytometry. The values above or below the bars indicate the corresponding percent inhibition relative to the allergen-stimulated, nonblocking mAb control. Percent inhibition (% inhibition) was calculated as: [1 — (sample — no stimulator)/(no blocking Ab control)] ′ 100. (**A** and **B**) Basophils sensitized with P1 (**A**) or P2 (**B**) serum and then stimulated with ACE. (**C**) Basophils sensitized with P1 serum and stimulated with AP-N. The anti-human IgE heavy chain Ab (α-IgE) was used as a positive control for basophil activation. VRC01LS IgG was used as a negative control. The P2 samples were run in triplicate (**B**). Due to the limited availability, P1 samples were run once in **A** and **C**.

The α-Gal allergenicity of ACE and AP-N was previously evaluated by the skin prick test and basophil activation test (BAT), which showed that ACE and AP-N at concentrations of 0.1–10 mg/ml activate basophils from patients with AGS, but not healthy individuals (53). To determine whether AG028 IgA_2_, AG050 IgA_1_, and AG028 IgM could inhibit AGS allergen-mediated basophil activation, we used an indirect BAT that quantifies the frequency of cells expressing the activation marker CD63^+^, as described previously (54). Basophils within PBMCs from healthy individuals were subjected to lactic acid stripping of membrane-bound IgE and sensitized with serum from 2 patients with AGS (P1, UNC0445.1 and P2, UNC0512.1). The sensitized cells were then stimulated with ACE or AP-N at a final concentration of 1mg/mL, based on a prior study reporting that this concentration gave near maximal activation with serum samples from 4 patients with AGS (53). CD63 expression on lineage-negative, HLA-DR negative, CD123/CD203c-positive basophils were assessed by flow cytometry in the presence or absence of AG028 IgA_2_, AG050 IgA_1_, and AG028 IgM (Figure 7 and Supplemental Figure 10A). VRC01LS, a human IgG_1_ mAb targeting the CD4-binding site of the HIV-1 envelope glycoprotein (55), does not bind to AGS allergens and was used as a negative control (Supplemental Figure 9).

Following stimulation with ACE alone, 55.7% of basophils sensitized with P1 serum (single replicate) were CD63^+^ (Supplemental Figure 10, B and C), and an average of 71.1% of basophils sensitized with P2 serum (triplicate) were CD63^+^ (Supplemental Figure 10D). Following stimulation with AP-N alone, 67.3% of basophils sensitized with P1 serum (single replicate) were CD63^+^ (Supplemental Figure 10, B and E). For basophils sensitized with P1 serum, both AG028 IgA2 and AG028 IgM inhibited ACE-mediated basophil activation, achieving 86.2% and 78.8% inhibition, respectively, at the highest concentration tested (Figure 7A). AG028 IgA_2_ and AG028 IgM inhibited AP-N–mediated basophil activation by 60.1% and 68.8%, respectively, at the highest concentration tested, which was lower than the inhibition observed for ACE-mediated activation (Figure 7C). AG050 IgA_1_ showed little effect on ACE- or AP-N-mediated basophil activation (Figure 7, A and C) despite near-complete blocking of polyclonal IgE binding to ACE in Figure 5B. For basophils sensitized with P2 serum, the 3 mAbs showed little inhibition of ACE-mediated basophil activation (Figure 7B).

To confirm that inhibition of basophil activation correlates with mAbs blocking IgE binding to ACE and APN, we repeated the IgE blocking assay using the same P1 and P2 sera (Supplemental Figure 11). With P1 serum, AG028 IgA_2_, AG050 IgA_1_, and AG028 IgM reduced IgE binding to ACE by approximately 30%, 40%, and 80%, respectively, with similar inhibition observed for APN. Interestingly, the AG028 clone showed higher functional inhibition regardless of Ig isotypes, IgA or IgM, or IgE blocking ability in P1 serum. In contrast, with P2 serum, only AG028 IgM partially inhibited IgE binding to ACE (45%). Collectively, IgE-binding blockade to ACE/APN by these blocking mAbs and BAT inhibition were not concordant across serum.

### Comparison of the sequences of α-Gal-specific mAbs isolated from malaria-exposed individuals versus patients with AGS.

**Figure 8:**
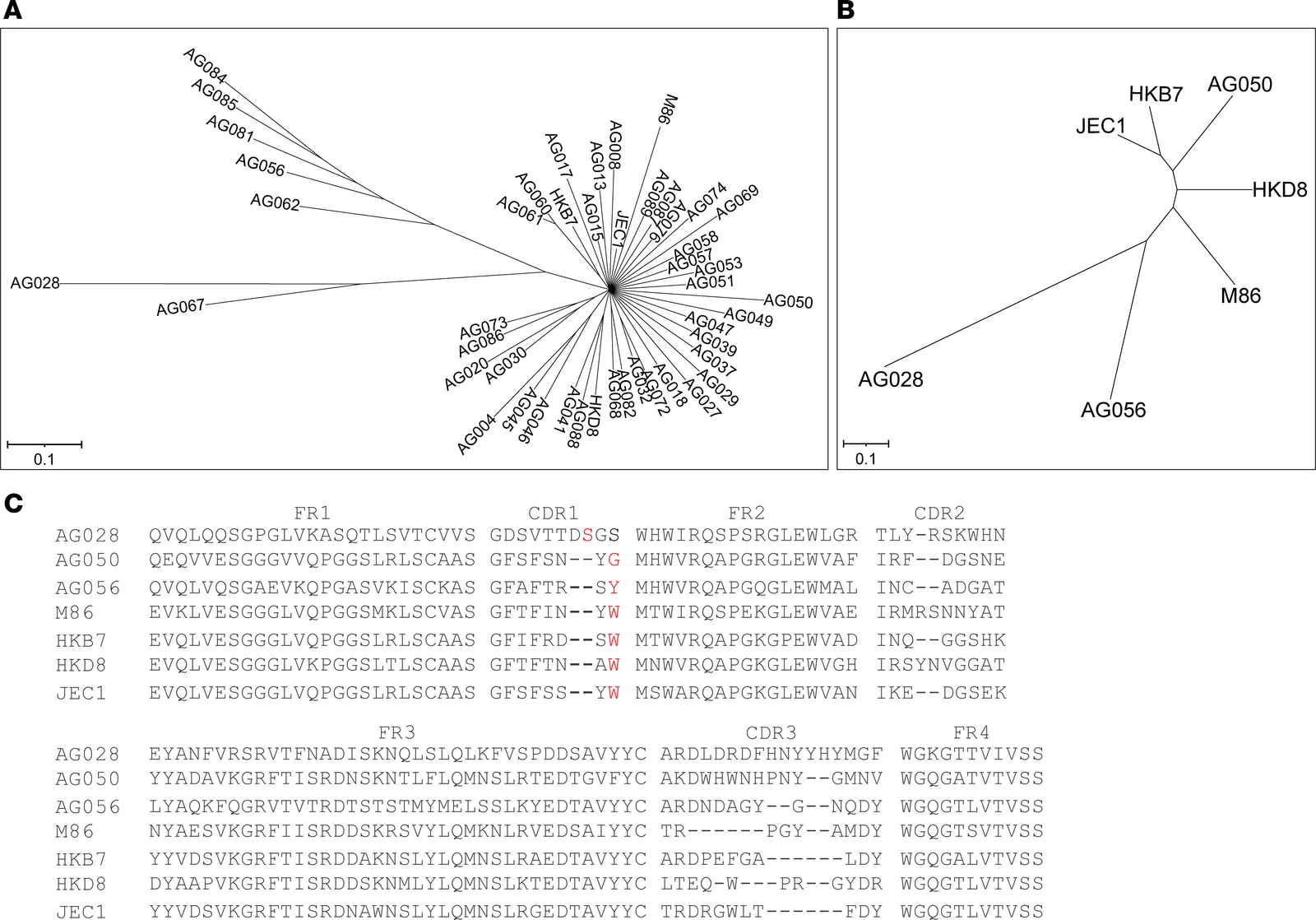
Comparison of VH sequences of α-Gal–specific mAbs derived from malaria-exposed individuals and patients with AGS. Abalign and Geneious Prime were used to align the VH sequences and to generate unrooted distance trees, respectively. (**A**) An unrooted distance tree that includes the 42 mAbs isolated from malaria-exposed individuals in this study (beginning with “AG”) along with the previously identified α-Gal–specific mAbs from patients with AGS (HKB7, HKD8, JEC1) and the murine mAb M86. (**B**) An unrooted distance tree that includes AG028, AG050, AG056, HKB7, HKD8, JEC1, and M86. (**C**) VH sequence alignment of AG028, AG050, AG056, HKB7, HKD8, JEC1, and M86, with CDRs shown in bold and residues at Kabat position 33 highlighted in red. FR; framework region.

Langley et al. reported approximately 400 heavy chain variable region (VH) sequences from single-sorted B cells of 4 individuals with a history of mammalian meat allergy, using AF647-conjugated α-Gal-BSA as a probe (41). Among these sequences, 3 antibodies—HKB7, HKD8, and JEC1—were confirmed for their expression and binding to α-Gal. To determine whether the 42 mAbs isolated from malaria-exposed individuals in this study exhibited any similarity to HKB7, HKD8, JEC1, and the murine mAb M86, we compared their VH sequences. Abalign, a sequence alignment platform for B cell receptor immune repertoire analysis (56), was used to align VH sequences. Subsequently, Geneious Prime was employed to generate an unrooted distance tree to illustrate the evolutionary relationships between VH sequences. Among the α-Gal–specific mAbs compared, the majority of mAbs isolated in this study clustered with the previously identified mAbs (Figure 8A). Notably, AG028, 1 of the 2 mAbs that interfered with serum IgE binding to AGS allergens, appeared on a long branch diverging from the main cluster of other VH sequences, indicating an evolutionary divergence from the main group of VH sequences (Figure 8A). Additionally, we compared the VH sequences of AG028 and AG050, both of which interfered with serum IgE binding to AGS allergens, and AG056, which did not, with those of HKB7, HKD8, JEC1, and M86. AG028 was the most divergent from other α-Gal antibodies in the unrooted distance tree (Figure 8B). The CDR3 length of AG028 was 17 amino acids, longer than the average CDR3 length of 13.1 observed across 42 α-Gal mAbs and substantially longer than the 4 reported α-Gal antibodies, which ranged from 9–11 amino acids (Figure 8C). Additionally, AG028 harbors serine at VH Kabat position 33, a residue that is relatively rare among α-Gal antibodies (40) (Figure 8C). These sequence characteristics of AG028 suggest that this antibody may utilize a distinct binding mechanism to interfere with serum IgE binding to AGS allergens.

## Discussion

The number of suspected cases of tick bite–associated AGS has increased substantially since 2010 (2), a trend potentially accelerated by climate-driven expansion of tick habitats (57). Currently, no disease-modifying therapy exists for this persistent and potentially life-threatening allergy (2). While symptoms may resolve in individuals who avoid further tick exposure, leading to a decline in α-Gal–specific IgE, clinical guidance on the safe reintroduction of mammalian-derived foods is limited (58). Omalizumab, an anti-IgE mAb has shown encouraging results in multiple food-allergy studies, either as monotherapy or in combination with allergen immunotherapy (AIT) (59), and has also been reported to impair α-Gal–dependent basophil activation triggered by α-Gal–containing glycolipid and cetuximab in vitro, highlighting the potential of antibody-based interventions (54). Furthermore, the development of allergen-specific mAbs to prevent allergic reactions to cat dander, birch pollen, and peanut allergy (30–34), provides a strong rationale for similar strategies in AGS.

To establish a translational framework for antibody-based therapies in AGS, we developed a multiplex bead–based assay to profile isotype- and antigen-specific antibody reactivities to α-Gal–bearing ligands and to prioritize clinically relevant antigen formats for mAb screening. Our findings from this assay indicate that IgE reactivity to ACE, AP-N, synthetic α-Gal, and cetuximab correlates with clinical diagnostic measurements of α-Gal–specific IgE in patients with AGS (Supplemental Table 3). Furthermore, IgE binding to ACE, APN, and cetuximab was largely α-Gal dependent, confirmed by the abrogation of plasma IgE binding to these proteins in the presence of excess soluble α-Gal, supporting α-Gal as the dominant IgE determinant on these allergens. However, we found that IgE reactivity of patients with AGS differed across α-Gal–bearing antigens. For example, although patients with AGS had elevated IgE reactivities to ACE, AP-N, and cetuximab compared with people who were healthy controls, this was not the case for milk proteins. This is consistent with our observation that most patients with AGS in this study reported no milk allergy (Supplemental Table 3), similar to what has been reported in larger cohorts of patients with AGS (45). It is possible that many patients with AGS do not have concurrent milk allergy because antibody recognition is shaped by the molecular context in which α-Gal is displayed (e.g., accessibility, epitope density/valency, and functional avidity) (50, 60, 61). Indeed, a recent study by Santra et al. suggests that variability in α-Gal immunogenicity may be related to differing degrees of rigidity and structural constraint across allergens (62). Therefore, α-Gal structural or conformational differences may contribute to variable levels of immunogenicity/allergenicity across α-Gal–containing allergens. Consistent with this hypothesis, our study provides indirect evidence of the existence of different α-Gal configurations by demonstrating differential antibody binding and competition patterns among synthetic α-Gal-BSA and naturally glycosylated α-Gal–containing proteins (ACE and AP-N). Additionally, it has been suggested that patients with AGS are less likely to be allergic to milk products because of a lower abundance of α-Gal epitopes on milk (63). The serologic analysis also revealed that α-Gal–reactive IgG levels showed weak-to-no correlation with α-Gal–specific IgE, despite elevated IgG_1–3_ to ACE, APN, and cetuximab in patients with AGS. This suggests that IgG responses may reflect exposure history and/or distinct immunologic pathways rather than mirroring the allergic IgE response, a finding consistent with studies showing that systemic IgG to dietary antigens is common in nonallergic individuals and correlates with intake (64). Together, the results of the multiplex bead-based assay provide insight into the serologic profiles of patients with AGS and offer a scalable screening platform for prioritizing clinically relevant α-Gal presentations to select candidate mAbs.

In this study, we initially aimed to identify mAbs targeting α-Gal on malaria parasites and isolated 42 α-Gal–binding mAbs from B cells of malaria-exposed individuals but found they bound weakly to *P*. *falciparum*. We then screened these mAbs for their ability to bind AGS allergens and found that 13 bound to ACE, AP-N, and cetuximab. An important result of this study is that only 2 of these mAbs, AG028 (expressed as IgA_2_ or IgM) and AG050 (IgA_1_) inhibited AGS patient IgE binding to ACE and AP-N, and only AG028 suppressed ACE-mediated activation of basophils sensitized with serum of patients with AGS. Several potential reasons may explain why all but one of the 42 α-Gal–binding mAbs were incapable of blocking the function of α-Gal–specific polyclonal IgE from patients with AGS. First, it is possible that the α-Gal–conjugated HSA probe we used for B cell sorting did not yield mAbs that consistently bind configurations of the α-Gal epitope on the AGS allergens used in this study. Second, the α-Gal–specific antibody repertoires of malaria-exposed individuals in Mali may differ from those of patients with AGS in North America due to differences in genetic background, immune history, and environmental exposures. Additionally, sequence analysis showed that AG028 is divergent from the other mAbs isolated in this study and other previously reported α-Gal–specific mAbs (HKB7, HKD8, JEC1, M86), with a longer CDR3 and a rare serine at VH Kabat position 33, suggesting that AG028 may utilize a distinct binding mechanism that underlies its unique functional activity.

Competition assays using allergen-coated beads indicated that AG028 and AG050 recognize overlapping features with M86 on bead-immobilized ACE, AP-N, and synthetic α-Gal. In contrast, AG056 bound synthetic α-Gal but neither competed with AG028 or AG050 nor blocked patient IgE binding to ACE or AP-N, supporting a model of context-dependent recognition of α-Gal across distinct carrier backbones. M86, generated against a natural α-Gal source, has also been reported to bind synthetic α-Gal and is thought to exhibit broad cross reactivity toward both natural and synthetic α-Gal epitopes (41, 52). On the contrary, AG056 showed weak binding to natural α-Gal sources, even though its binding to synthetic α-Gal was comparable with that of AG050 IgA1. Together, these findings suggest that AG028 and AG050 recognize broadly cross-reactive α-Gal epitopes, similar to M86, rather than epitopes with a preference for synthetic α-Gal. Notably, bead-based competition assays correlated well with Fab binding affinities to α-Gal epitopes measured by BLI, suggesting that binding affinity is more predictive of competition outcomes than of serum IgE blocking activity.

Also of note is the observation that AG028 inhibited ACE- and AP-N–induced basophil activation in a donor-dependent manner, possibly reflecting heterogeneity in polyclonal IgE affinity, and/or differences in epitope distribution or α-Gal presentation on ACE and APN in vivo. Interestingly, the bead-based IgE competition results did not consistently predict functional inhibition in the iBAT assay, possibly due to differences in assay configuration (immobilized versus solution-phase allergen presentation) and/or the complex biological thresholds governing basophil activation. That is, the bead assay primarily reports epitope occupancy on a solid phase, whereas iBAT inhibition requires preventing FcεRI crosslinking on the cell surface, a process sensitive to ligand valency, receptor density, and signaling thresholds (60, 61).

Interestingly, the most effective inhibitory mAbs in our study were IgA isotypes. While IgG is known to suppress basophil activation via FcγRIIB engagement (65), IgA has also been reported to inhibit IgE-mediated activation of mast cells and basophils in an allergen-specific manner, and AIT can induce substantial allergen-specific IgA responses at mucosal sites (28). The potent functional activity of AG028 as an IgA_2_ in the P1 serum, despite its modest performance in bead-based competition assays compared with its IgM counterpart, suggests a mechanism beyond simple epitope blocking. This may involve IgA isotype-dependent inhibitory signaling pathways that warrant further investigation.

The mAbs generated from memory B cells in this study exhibited modest apparent affinities compared with food allergen–specific mAbs previously developed for IgE-mediated diseases (34, 66). Glycans can elicit relatively low-affinity antibody responses due to limited immunogenicity and structural similarity among carbohydrate epitopes (67). Although carbohydrate-specific antibodies have traditionally been considered largely T cell independent (68), high-affinity carbohydrate-specific antibodies can arise under some conditions (69). Because high-affinity antibodies are likely required for more effective neutralization of allergen-specific IgE (70), future efforts will focus on isolating more potent mAbs from patients with AGS or improving affinity through protein engineering such as high-affinity allergen-specific antibodies reexpressed as IgG (71), or affinity enhancement by nondeleterious CDR mutations guided by computational approaches (72).

Going forward, the approaches described here could be used to isolate potent neutralizing/blocking mAbs from plasmablasts and memory B cells of patients with AGS and to identify antibodies that block IgE binding across multiple α-Gal–bearing allergens. Using different α-Gal–bearing probes for B cell sorting and screening may broaden the spectrum of blocking activity. Because human IgE memory B cells are rare (73) and IgG memory B cells can rapidly class switch to IgE upon reexposure and expand into high-affinity plasmablasts (73, 74), comparing antibodies derived from plasmablasts versus memory B cells may clarify how α-Gal antibody responses evolve and how they relate to clinical phenotypes. As noted above, it will also be of interest to assess whether and how B cell clones isolated from patients with AGS differ from those isolated from individuals in the Mali cohort.

In conclusion, this study provides proof-of-concept that α-Gal–specific mAbs can inhibit IgE binding to key AGS allergens and attenuate downstream cellular activation. These findings support continued efforts to discover and develop targeted antibody-based therapeutics for AGS.

## Methods

### Sex as a biological variable.

Both the AGS patient cohort and the cohort of malaria-exposed individuals in Mali included male and female participants. However, sex was not considered as a biological variable in the analyses.

### Measuring total and α-Gal specific IgE levels in the AGS patient.

On the day of enrollment, all participants had venous blood drawn into serum separator tubes. All whole blood samples were separated into serum by centrifugation within 24 hours of collection and stored at 4°C for no more than 24 hours before further processing. Total and α-Gal specific IgE antibodies were measured using commercially available ImmunoCAP (Code o215, Phadia ThermoFisher US). The assays were performed with the ImmunoCAP 250 instrument, and the results were expressed as IU per milliliter, where the one IU both for total and α-Gal specific IgE is approximately 2.4 ng. Sera were assayed for sIgE to α-Gal per manufacturer instructions and the cutoff for a positive test was the limit of detection, 0.1 IU/mL.

### Measuring α-Gal–specific Ab responses in the Malian cohort.

The levels of α-Gal–specific IgM, IgG, and IgA in plasma samples from the Mali cohort were determined using a multiplex bead assay. Synthetic α-Gal linked to BSA (Dextra, Cat# NGP0334), bovine thyroglobulin (Sigma-Aldrich, Cat# 609310), and human thyroglobulin (Sigma-Aldrich, Cat# 609312) were biotinylated using a biotin conjugation kit (Abcam, Cat# ab201796) and captured on streptavidin-coated beads with different intensities of phycoerythrin (PE)-channel fluorescence (Spherotech, Cat# SVFA-2558-6K and SVFB-2558-6K). Each antigen was coupled to beads in a single batch, aliquoted, frozen, and used throughout the study to minimize batch effects. All antigen-coupled bead populations were incubated together with plasma sample in a single well for antibody binding and plasma antibodies bound to bead-coupled antigens were detected using AF647-conjugated anti-human IgM, IgG, or IgA pAbs (Jackson Immunoresearch, Cat# 109-606-129, 109-606-170, and 109-606-011, respectively). To minimize batch effects, the same lot of AF647-conjugated secondary antibody was used throughout the study. PBS containing 0.5% BSA was used as the assay buffer throughout. Plates were read on an iQue Screener Plus high-throughput flow cytometer (Sartorius). Antibody binding was quantified as AF647 MFI, compensated for bead fluorescence in the PE channel, and background-subtracted using human thyroglobulin as an internal negative control. Flow cytometry data were analyzed using FlowJo (BD Biosciences).

### Cell lines.

Expi293F and ExpiCHO-S cells were obtained from Thermo Fisher Scientific (Cat# A14527 and A29127, respectively). Cells were cultured in Expi293 Expression Medium (Gibco, Cat# A1435101) or ExpiCHO Expression Medium (Gibco, Cat# A2910001) on an orbital shaker in a 37°C incubator with 8% CO_2_ and ≥ 80% relative humidity.

### Isolation, sequence analysis, and production of anti-α-Gal mAbs.

To isolate anti-α-Gal mAbs, 18 individuals were selected from a cohort of approximately 500 individuals in Mali based on high anti-α-Gal antibody titers. Cryopreserved PBMCs of these individuals were thawed and stained with LIVE/DEAD Fixable Aqua (ThermoFisher Scientific, Cat# L34965). Gala1-3Galb1-4GlcNAc-HSA and HSA were purchased from Dextra (Cat# NGP2334) and Sigma-Aldrich (Cat# A3782) and conjugated with biotin and APCCy7 (Novus, Cat# 765-0005), respectively. PBMCs were then incubated with biotinylated α-Gal-HSA and HSA-APCCy7 in 0.5% HSA in PBS at 4°C for 45 minutes. Subsequently, cells were washed and stained with the following panel: CD14-BV510 (BioLegend, Cat# 301842, clone M5E2), CD3-BV510 (BioLegend, Cat# 317332, clone OKT3), CD56-BV510 (BioLegend, Cat# 318340, clone HCD56), CD19-PECy7 (BioLegend, Cat# 115520, clone 6D5), IgD-FITC (BioLegend, Cat# 348206, clone IA6-2), IgA-DyLight405 pAb (Jackson Immunoresearch, Cat# 109-475-011), IgM-PerCP-Cy5.5 (BioLegend, Cat# 314512, clone MHM-88), PE-conjugated Streptavidin (BioLegend, Cat# 405204), and AF647-conjugated Streptavidin (BioLegend, Cat# 405237), and sorted using BD FACSAria (BD Biosciences). Sorted IgM^+^, IgA^+^, and IgM^–^IgA^–^ (IgG^+^) B cells were plated 1–2 cells/well into 384-well plates containing IMDM medium (Gibco, Cat# 31980-030) with 10% FBS, 100 U/mL of IL-2 (Roche, Cat# 11147528001), 50 ng/mL of IL-21 (ThermoFisher Scientific, Cat# PHC0211), and ~5,000 irradiated 3T3-CD40L cells per well. Following 2 weeks of cell culture, supernatants were harvested and screened for α-Gal binding. DyLight405-conjugated goat anti-human IgA (Jackson Immunoresearch, Cat# 109-475-011), AF647-conjugated IgG (Jackson Immunoresearch, Cat# 109-606-170), and AF647-conjugated IgM (Jackson Immunoresearch, Cat# 109-606-129) polyclonal secondary antibodies were used for detection and plates were read with the iQue Screener Plus high-throughput flow cytometer. FACS data were analyzed using the Forecyt program (Sartorius).

From the B cell clones identified as positive for α-Gal binding, cDNA was synthesized followed by PCR-amplification and immunoglobulin heavy and light chains were sequenced. Sequences were analyzed using the IMGT database (39) to determine VH and VL genes and the percentage of somatic mutations. Each VH and VL gene of 4 IgG and 31 IgM was synthesized after codon optimization, cloned into a human IgG_1_ expression plasmid, and expressed in CHO cells (GenScript). Seven IgA genes were cloned into their original IgA_1_ or IgA_2_ expression plasmid and IgA mAbs were expressed without J chain in CHO cells (GenScript). For further mAb production, expression plasmids were transfected into Expi293F cells (ThermoFisher Scientific, Cat# A14527) using Polyethylenimine Max transfection reagent (Polysciences Inc, Cat# 24765) and IgG_1_ and IgA_1/2_ mAbs were purified by high-performance liquid chromatography using HiTrap Protein A HP antibody-purification columns (Cytiva, Cat# 17040303) and CaptureSelect IgA Affinity Matrix (ThermoFisher Scientific, Cat# 194311005), respectively. Some of the original IgM clones made as IgG_1_ were re-cloned into a human IgM expression vector. IgM plasmids with J chain were transfected into ExpiCHO-S cells (ThermoFisher Scientific, Cat# A29127) using the ExpiFectamine CHO Transfection Kit (Gibco, Cat# A29129). Eighteen hours post-transfection, enhancer and feed were added, and the temperature was lowered to 32°C with 5% CO_2_ according to the High Titer protocol, following the manufacturer’s instructions. The mAbs were purified using the POROS CaptureSelect IgM Affinity Matrix (ThermoFisher Scientific, Cat# 2812892005).

### α-Gal-specific mAb binding to Plasmodium sporozoites and CSP antigens.

Live *P*. *falciparum* sporozoites isolated from the salivary glands of *An*. *gambiae* or *An*. *stephensi* mosquitoes, or live *P*. *falciparum* CSP-containing transgenic *P*. *berghei* sporozoites isolated from the salivary glands of *An*. *stephensi*, were incubated with anti-α-Gal mAbs in 96-well plates for 30 minutes at 4°C. After washing, sporozoites were stained with AF647-conjugated anti-human IgM or IgA pAbs (Jackson Immunoresearch, Cat# 109-606-129 and 109-606-011, respectively). Plates were analyzed by flow cytometry, and data were processed as described above.

Biotinylated recombinant *P*. *falciparum* CSP and peptide antigens containing various regions of CSP were captured on PE-channel streptavidin beads as previously described (43). Antigen-coated beads were incubated with AG062 IgM for 30 minutes at room temperature (RT), washed, and stained with AF647-conjugated anti-human IgM pAb (Jackson Immunoresearch, Cat# 109-606-129). Plates were analyzed by flow cytometry, and data were processed as described above.

### AGS patient serum and mAb binding to AGS allergen.

α-Gal–containing allergens were purchased; ACE from porcine kidney (Sigma, Cat# A2580), AP-N from porcine kidney (Sigma, Cat# L9776), cetuximab (purchased through the UNC Shared Services Pharmacy), lactoferrin from bovine milk (Sigma, Cat# L9507), lactoperoxidase from bovine milk (Sigma, Cat# L2005), bovine gamma globulin, purified, fraction II, (ThermoFisher Scientific, Cat# 23212), gelatin from bovine skin, type B (Sigma, Cat# G9391), and gelatin from porcine skin, type A (Sigma, Cat# G1890). These allergens were biotinylated using a biotin conjugation kit (Abcam, Cat# ab201795) and captured on streptavidin-coated beads with different intensities of FITC- or PE-channel fluorescence as previously described (43). For IgG_1_ binding assays, biotinylated cetuximab was processed to generate F(ab′)_2_ fragments lacking the Fc region, thereby preventing recognition by anti-human IgG_1_ secondary antibodies, using a preparation kit (ThermoFisher Scientific, Cat# 44988). The approximately 110 kDa F(ab′)_2_ fragments were isolated by size-exclusion chromatography using a column (Cytiva, Cat# 28990944) and subsequently conjugated to streptavidin-coated beads. These antigen beads were incubated with α-Gal mAbs (serially diluted in 1:4) or AGS patient serum samples (1:10 dilution) in 96-well plates for 30 minutes at RT, washed, and stained with 2.5 μg/mL of APC-conjugated goat anti-human IgE pAb (Abcam, Cat# ab99898), AF647-conjugated anti-human IgA pAb (Jackson Immunoresearch, Cat# 109-606-011), or AF647-conjugated mouse anti-human IgG_1_, IgG_2_, IgG_3_, or IgG_4_ (Southern Biotech, Cat# 9052-31, 9070-31, 9210-31, 9200-31; clone 4E3, HP6002, HP6050, and HP6025, respectively). Plates were analyzed by flow cytometry, and data were processed as described above.

### Assessment of soluble α-Gal inhibition of α-Gal-specific Ab binding.

To assess the effect of soluble α-Gal on α-Gal–specific Ab binding, α-Gal mAbs or serum samples were incubated with or without 10 mM α-Gal trisaccharide (Dextra, Cat# L330) for 30 minutes at RT, followed by incubation with antigen-coated beads for an additional 30 minutes at RT. Binding of α-Gal mAbs was detected using AF647-conjugated anti-human IgM or IgA pAbs (Jackson Immunoresearch, Cat# 109-606-129 and 109-606-011, respectively). Serum IgE binding was detected using APC-conjugated goat anti-human IgE pAb (Abcam, Cat# ab99898). Plates were analyzed by flow cytometry, and data were processed as described above.

### Competition assay of anti-α-Gal mAbs for AGS allergen binding.

AGS allergen-coated beads were pre-incubated with unlabeled anti-α-Gal mAbs at increasing concentrations (0.01 ng/mL to 100 μg/mL in 1:4 serial dilutions) for 30 minutes at RT. Anti-α-Gal IgAs were conjugated with AF647 using an AF647-conjugation kit (Abcam, Cat# ab269823). Subsequently, AF647-conjugated AG028 IgA_2_ (1 μg/mL) or AF647-conjugated AG050 IgA_1_ (4 μg/mL) were added without washing, and the mixtures were incubated for an additional 30 minutes at RT. After washing, plates were analyzed by flow cytometry, and data were processed as described above. M86 mouse IgM was purchased as hybridoma culture supernatant (Enzo, Cat# ALX-801-090). The concentration of M86 antibody in the supernatant was determined using an IgM Mouse Uncoated ELISA Kit (Invitrogen, Cat# 88-50470) according to the manufacturer’s instructions, yielding a concentration of 0.93 μg/mL.

### Biolayer interferometry kinetic binding assay.

Antibody binding kinetics were measured using biolayer interferometry on an Octet R8 instrument (Sartorius). Fab mAbs were expressed in CHO-S cells with a C-terminal His tag on the heavy chain and purified using AmMag Ni Magnetic Beads, achieving ≥ 97% purity as determined by HPLC. Biotinylated AGS allergens were immobilized on streptavidin biosensors (Sartorius, Cat# 18-5136) for 300 seconds, followed by equilibration in HBS-EP+ buffer (Cytiva, Cat# BR100669) for 60 seconds to assess baseline drift. Association was measured by dipping the biosensors into serial dilutions of anti-α-Gal mAbs (3.125–400 nM for IgA/M and 0.75–48 μM for Fab) for 60 seconds, followed by dissociation in buffer. Background subtraction of nonspecific binding was performed using reference biosensors that underwent the same steps except allergen loading. Data analysis and curve fitting were performed using Octet BLI Analysis software, version 12.2.2.4. Experimental data were fitted with the binding equations describing a 1:1 analyte-ligand interaction.

### Interference of anti-α-Gal mAbs with AGS serum IgE binding to AGS allergens.

Anti-α-Gal mAbs (50 mg/mL final concentration) were incubated with AGS allergen-coated beads for 30 minutes at RT. AGS serum samples with the highest IgE reactivities (UNC0146.2, UNC0147.1, UNC0209.1, and UNC0383.1; 1:4 final dilution) were then added to the mixture without washing and incubated for 30 minutes at RT. After washing, APC-conjugated goat anti-human IgE pAb (Abcam, Cat# ab99898) was added and incubated for 30 minutes at RT. Plates were analyzed by flow cytometry, and data were processed as described above.

### Comparison of the VH sequences of antibodies.

We utilized Abalign (56) to align the VH region sequences of multiple antibodies. Subsequently, Geneious Prime (Geneious Prime 2024.0.3) was employed to generate unrooted distance trees.

### Indirect basophil activation assay.

Basophils within PBMCs isolated from healthy individuals were stripped of IgE with cold lactic acid, washed with RPMI-1640 containing 1% autologous serum, and then sensitized with the serum of patients with AGS overnight as previously described (54). Anti-α-Gal mAbs (1:4 titration from 200 to 3.125 μg/mL) were preincubated with 1 μg/ml of ACE or AP-N from the porcine kidney in RPMI-1640 containing 1% autologous serum and IL-3 (2 ng/mL) for 30 minutes at RT and then added to sensitized cells and incubated for 30 minutes at 37°C for basophil activation. Polyclonal rabbit anti-human IgE heavy chain Ab (Bethyl Laboratories Inc, Cat# A80-109A) at 1 μg/ml was used as a positive control. Stimulation reactions were stopped with 2.5 mM EDTA, and cells were stained for flow cytometry using the anti-human antibodies, including CD123-BV421 (BD Biosciences, Cat# 562517, clone 9F5), lineage cocktail-1 FITC including CD3 (clone SK7), CD16 (clone 3G8), CD19 (clone SJ25C1), CD20 (clone L27), CD14 (clone MφP9), and CD56 (clone NCAM16.2) mAbs (BD Biosciences, Cat# 340546), HLA-DR-PerCp-Cy5.5 (ThermoFisher Scientific, Cat# 45-9956-42, clone LN3), CD63-APC (BioLegend, Cat# 353008, clone H5C6) and CD203c-PE (Beckman Coulter, Cat# IM3575, clone 97A6) in staining buffer (2% FBS, 0.02% sodium azide in PBS). The samples were run on an Attune NxT flow cytometer (ThermoFisher Scientific) and data were analyzed using FlowJo v10.9.0. Percentages of CD63-positive basophils (lineage-1^–^, HLA-DR^–^, CD123^+^, CD203c^+^) were determined and used as a marker of basophil activation.

### Statistics.

Mutation frequencies among IgG, IgA, and IgM isotypes were compared using the Kruskal–Wallis test followed by Dunn’s post hoc multiple-comparisons test. A *P* value of less than 0.05 was considered statistically significant. Serum antibody responses among the αGal–specific IgE groups and healthy controls were compared using 1-way ANOVA followed by multiple-comparisons testing. *P* values of less than 0.05 were considered statistically significant (*P < 0.05, **P< 0.01, ***P < 0.001, and ****P < 0.0001). Correlation analyses were performed using JMP software. Associations between α-Gal–specific IgE levels, allergen-specific IgE MFIs, and IgG subclass MFIs were evaluated by linear regression. Coefficient of Determination (R^2^) and *P* values were calculated for each comparison. Only statistically significant regressions with R² > 0.05 are presented. IC_50_ values were calculated using GraphPad Prism software by fitting the competition binding data to a 4-parameter logistic sigmoidal model, with antibody concentration as the independent variable. Only curves with R² > 0.95 were considered reliable and are reported; curves with R² < 0.95 were designated as not determined (ND).

### Study approval.

The AGS studies reported here were approved by the Institutional Review Boards (IRBs, approval number 16-1533), Office of Human Research Ethics (OHRE) at the University of North Carolina (UNC), Chapel Hill under approval number 16-1533. Patient serum samples were collected at the UNC Allergy and Immunology Clinic with their consent.

Plasma and PBMC samples of malaria-exposed individuals were obtained from a cohort study conducted in Kalifabougou, Mali, which was approved by the Ethics Committee of the Faculty of Medicine, Pharmacy and Dentistry at the University of Sciences, Technique and Technology of Bamako, and the Institutional Review Board of the National Institute of Allergy and Infectious Diseases, National Institutes of Health (NIH IRB protocol number: 11IN126; ClinicalTrials.gov number: NCT01322581). Written informed consent was obtained from participants or the parents or guardians of participating children before inclusion in the study.

### Data availability.

All data associated with this study are in the paper or supplementary materials. The sequences of the antibody heavy and light chains generated in this study have been deposited in GenBank under accession numbers PZ582914-PZ582997. Values for all data points shown in graphs are provided in the Supporting Data Values file.

## Author contributions

HC conceived and designed the study, isolated and characterized mAbs, conducted most experiments, acquired and analyzed data, and wrote and edited the manuscript. BT and PDC designed and supervised the conduct of the Mali cohort study. YS prepared mAbs for some experiments, conducted the bead assays and the biolayer interferometry assay, analyzed data, and edited the manuscript. HS edited the manuscript. SKC performed the basophil activation assay. JS conducted statistical analysis, and MZ column-purified mAbs. LK assisted B cell sorting, and WZ assisted preparing antigens for the bead assays. JL conducted sequence comparison, and SL managed the biospecimens. JT, SPC, and PDC provided scientific expertise and critical reading of the manuscript. PDC wrote and edited the manuscript and provided funding.

## Conflict of interest

HC, JT, and PDC have submitted Provisional U.S. Patent Application No. 63/647,859, filed in February 2024, describing human anti-α-Gal antibodies.

## Funding support

This work is the result of NIH funding, in whole or in part, and is subject to the NIH Public Access Policy. Through acceptance of this federal funding, the NIH has been given a right to make the work publicly available in PubMed Central.

- The Intramural Research Program of the National Institutes of Health (NIH).
- NIAID/NIH grant R01 AI135049 (SPC).

## Supplementary Material

Supplemental data

Supporting data values

## Version 1 09/01/2026

Electronic publication
