# Supplementary material for: Human monoclonal antibodies targeting α-Gal restrict IgE engagement of α-Gal syndrome allergens
Source: J Clin Invest. 2026 Sep 1;136(17):e192370. doi: 10.1172/JCI192370 (PMC13528919; doi:10.1172/JCI192370)
Supplement: Supplementary file 1 [file jci-136-192370-s325.pdf]

Supplemental Figures and Tables for

Human monoclonal antibodies targeting α-Gal restrict IgE engagement of α-Gal syndrome allergens

Hyeseon Cho, Youngsil Seo, Haewon Sohn, Shailesh K. Choudhary, Jeff Skinner, Ming Zhao, Ludmila Krymskaya, Weizhi Zhong, Justin Lack, Shanping Li, Boubacar Traore, Joshua Tan, Scott P. Commins, Peter D. Crompton

Corresponding authors

Scott P. Commins, Phone: 919-537-3306; Email: scommins@email.unc.edu

Peter D. Crompton, Phone: 301-761-5042; Email: pcrompton@niaid.nih.gov

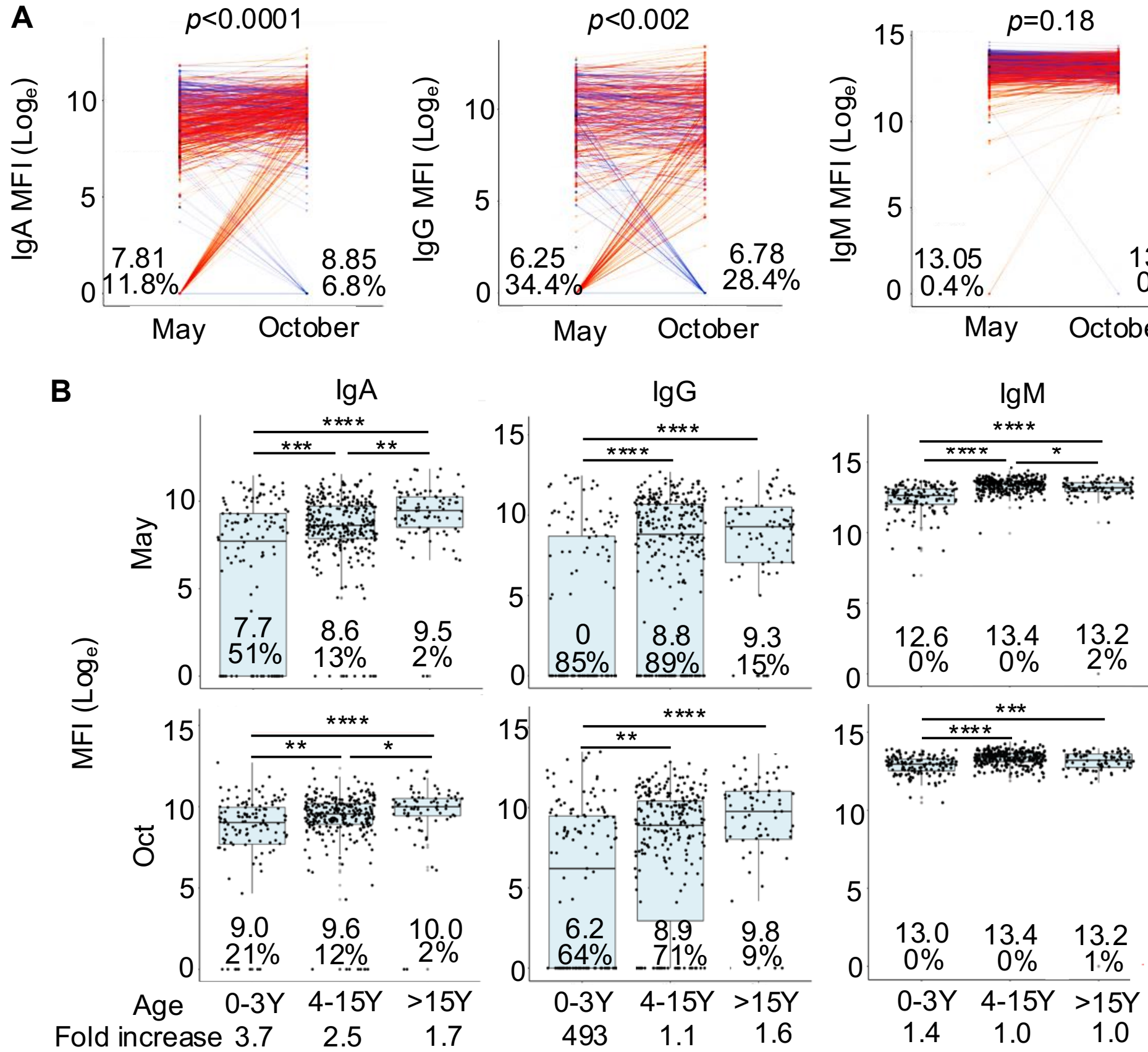

Supplemental Figure 1. Anti-α-Gal IgA, IgG, and IgM responses of Malian cohort. (A) Paired analysis to observe seasonal changes in IgA, IgG, and IgM responses to α-Gal. Plasma samples were collected in May, the end of the dry season (n=550), and in October, at the peak of malaria transmission (n=503). MFI: median fluorescence intensity. The paired median MFIs and percentages of individuals with no detectable antibody responses are shown above the X-axis. The Y-axis is plotted in natural log scale. The red and blue lines represent participants with higher or lower MFIs in October compared to May, respectively. p-values are from a mixed-effects ANOVA. **(B)** Antibody responses to α-Gal in the same study participants stratified into three age groups in May (0-3 years, n=140; 4-15 years, n=317; >15 years n=93) and October (0-3 years, n=139; 4-15 years, n=286; >15 years, n=78). The median MFIs and percentages of individuals with no detectable antibody responses in each age group are shown above the X-axis. Fold increases in MFI from May to October in each age group are shown below the X-axis. The Y-axis is plotted in natural log scale. p-values are from Bonferroni-adjusted Dunn's tests. * $p<0.05$; ** $p<0.01$; *** $p<0.001$; **** $p<0.0001$. Synthetic α-Gal linked to BSA was used to measure α-Gal-specific Ab responses in a buffer containing 0.5% BSA in PBS.

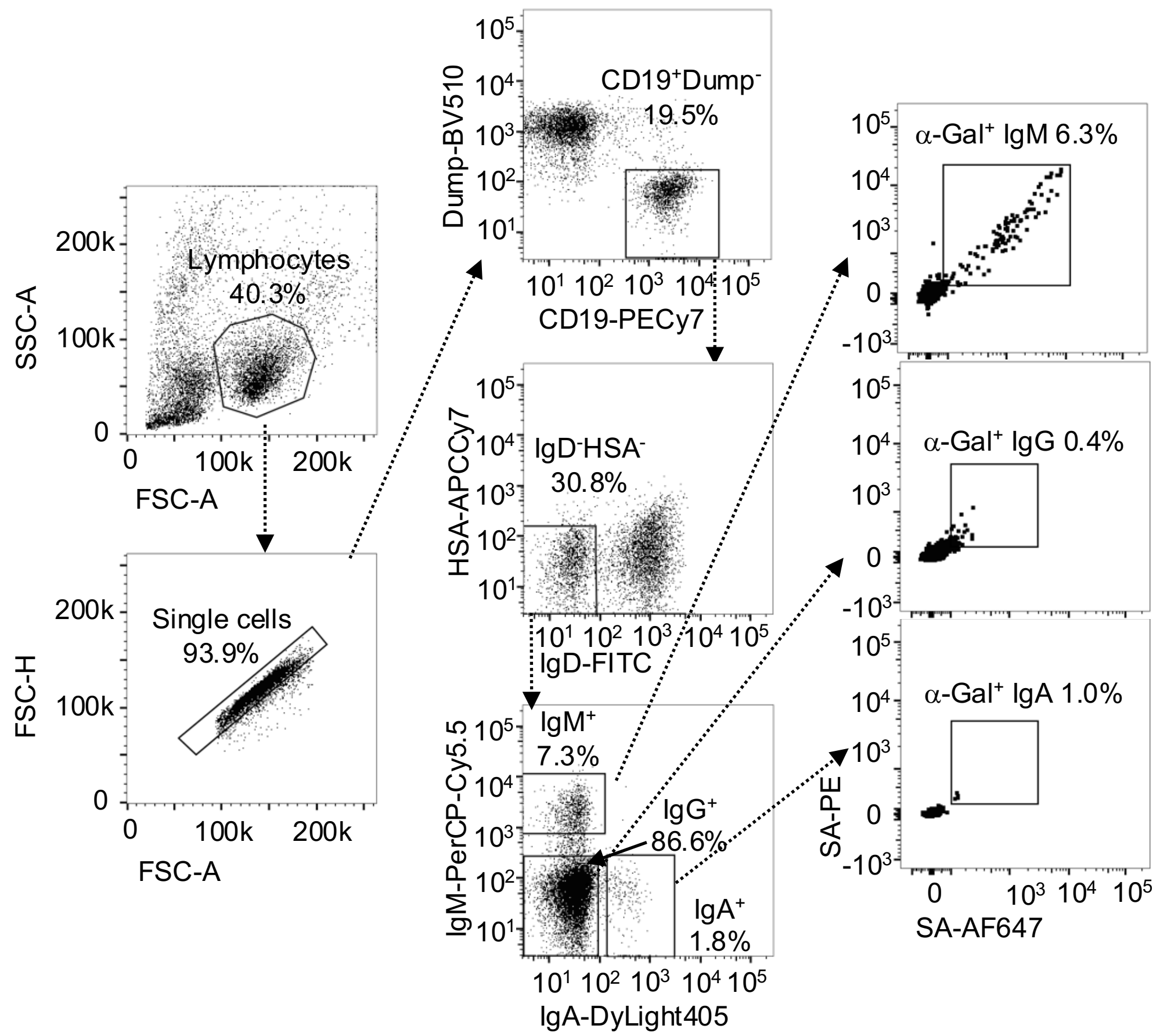

Supplemental Figure 2. Fluorescence-activated cell sorting strategy. A representative flow cytometry plot. HSA conjugated with an average of 27 synthetic α-Gal molecules was biotinylated and used to isolate α-Gal-binding B cells from PBMCs as described in Methods. $CD19^+$ B cells were gated into three populations (IgM^+, IgA^+, and IgM^-IgA^-) after gating out dead cells, innate cells, T cells, IgD^+ B cells, and HSA-APCCy7-binding B cells. Streptavidin (SA)-PE and SA-Alexa Fluor 647 double positive cells were sorted and cultured for B cell activation.

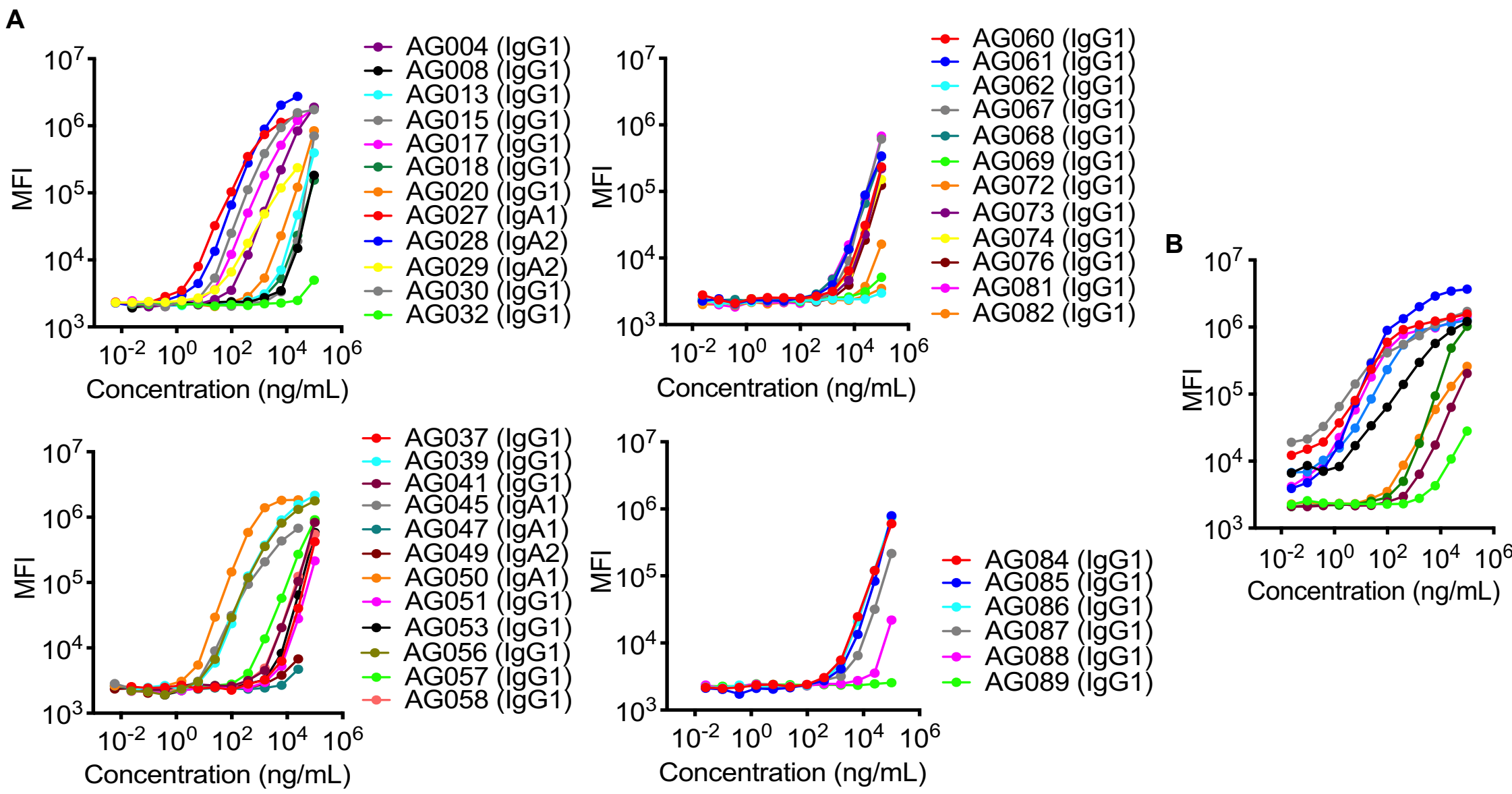

Supplemental Figure 3. Anti-α-Gal recombinant mAb binding to synthetic α-Gal-conjugated to BSA beads. (A) Titration curves (1 to 4) of 42 anti-α-Gal IgG_1, IgA_1, and IgA_2 mAbs without J chain. The starting concentrations were 250 µg/mL for IgG_1 and 50 µg/mL for IgA_1 and IgA_2. **(B)** Ten clones (7 clones initially isolated as IgM; one IgG clone and two IgA clones selected based on their binding activities to various α-Gal-expressing pathogens) were expressed as IgM with J chain. Titration curves (1 to 4) of 10 anti-α-Gal IgM with J chain was performed with the starting concentration of 100 µg/mL.

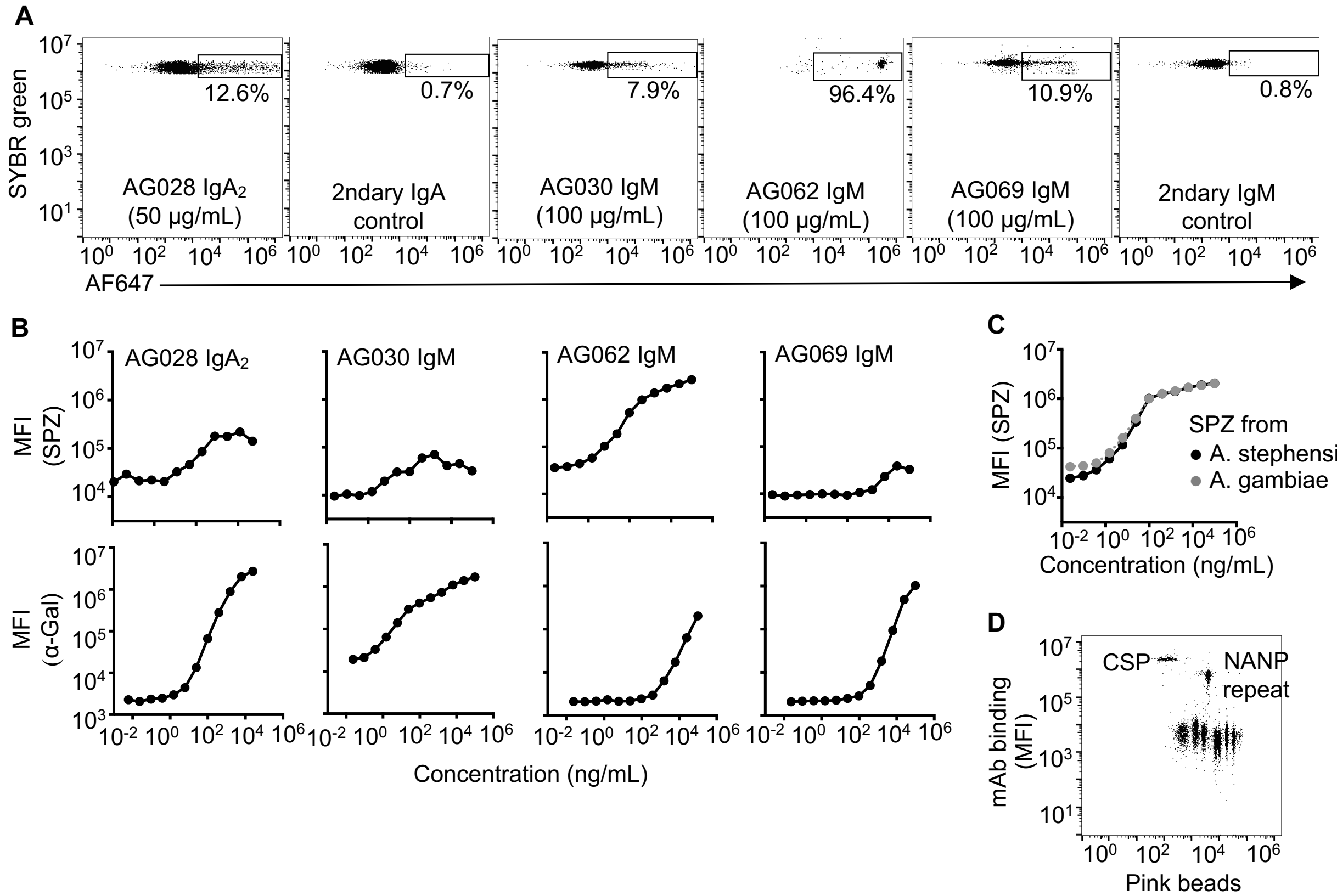

Supplemental Figure 4. Binding of anti-α-Gal mAbs to *Plasmodium* sporozoites. (A) Representative flow cytometry plots of the top four anti-α-Gal mAb binding to live *P. berghei* sporozoites transgenic for *Pf*CSP that had been isolated from *Anopheles stephensi*. **(B)** Titration curves (1:4 dilution) of the top four anti-α-Gal mAb binding to live *Pf*CSP transgenic *P. berghei* sporozoites (top panel) and α-Gal beads (bottom panel). **(C)** Titration curve (1:4 dilution) of AG062 mAb binding to live *P. falciparum* sporozoites isolated from *A. stephensi* (black) and *A. gambiae* (grey). **(D)** Binding of AG062 IgM to streptavidin-conjugated pink beads coated with various regions of *Pf*CSP. Pink bead 1 (P1): full-length *Pf*CSP, P3: P18 peptide, P5: P21 peptide, P6: N-terminal domain of *Pf*CSP, P7: NANP repeat, P8: C-terminal domain of *Pf*CSP, P9: CD4 negative control, P10: *P. vivax* region I, P12: blood stage MSP-1 protein (1).

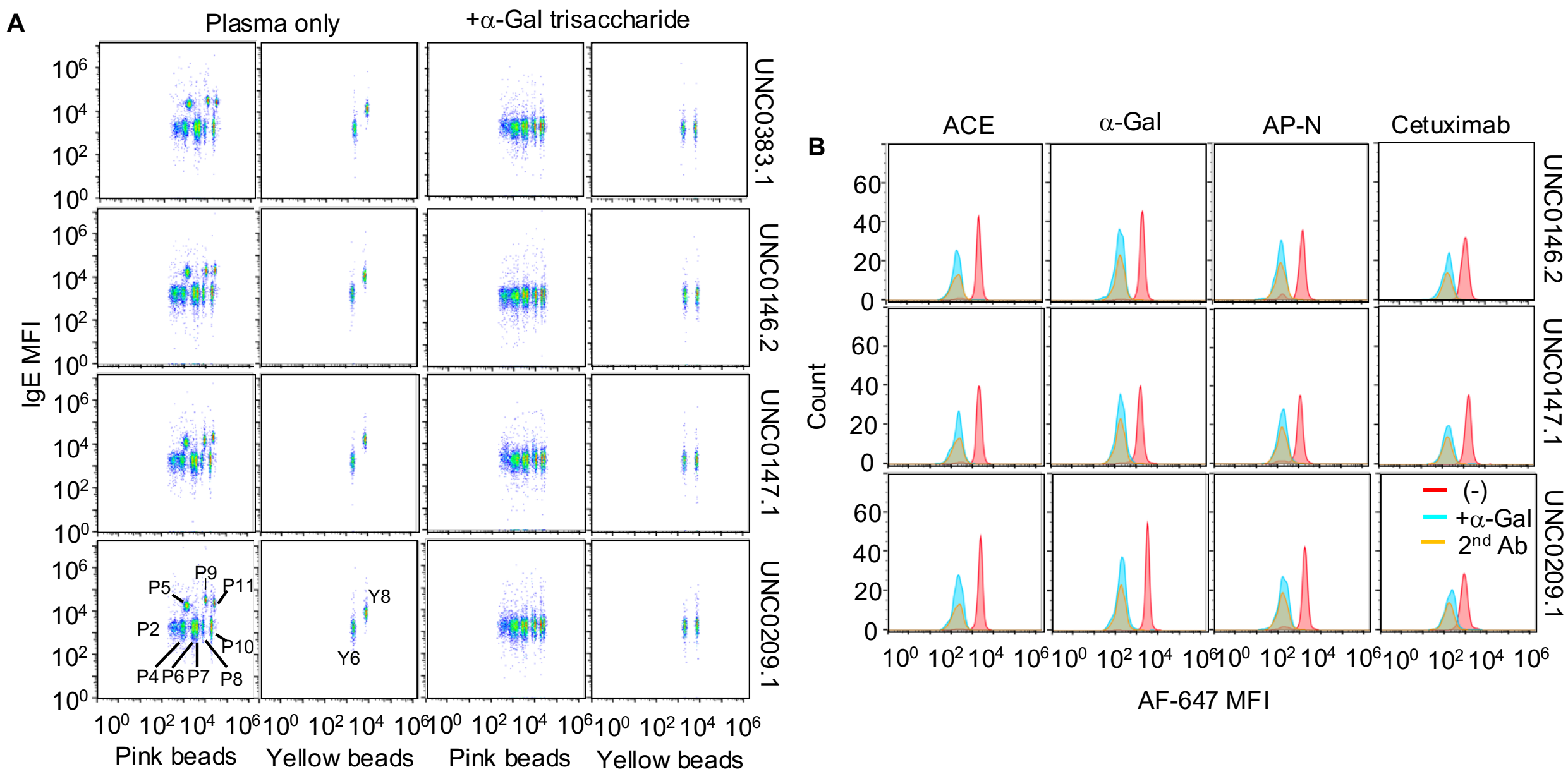

Supplemental Figure 5. Multiplex AGS allergen bead assay with plasma of AGS patients. (A) Flow cytometry plots of four AGS patient plasma samples (UNC0383.1, UNC0146.2, UNC0147.1, UNC0209.1) in the presence and absence of 10 mM α-Gal trisaccharide. Pink beads: P2-bovine gelatin, P4-LF, P5-AP-N, P6-BGG, P7-human thyroglobulin (negative control), P8-porcine gelatin, P9-α-Gal, P10-LPO, P11-ACE. Yellow beads: Y6-human thyroglobulin (negative control), Y8-Cetuximab. **(B)** Overlay of three flow cytometry plots: serum only (-), plus 10 mM α-Gal trisaccharide (+α-Gal), and secondary Ab only control (2nd Ab).

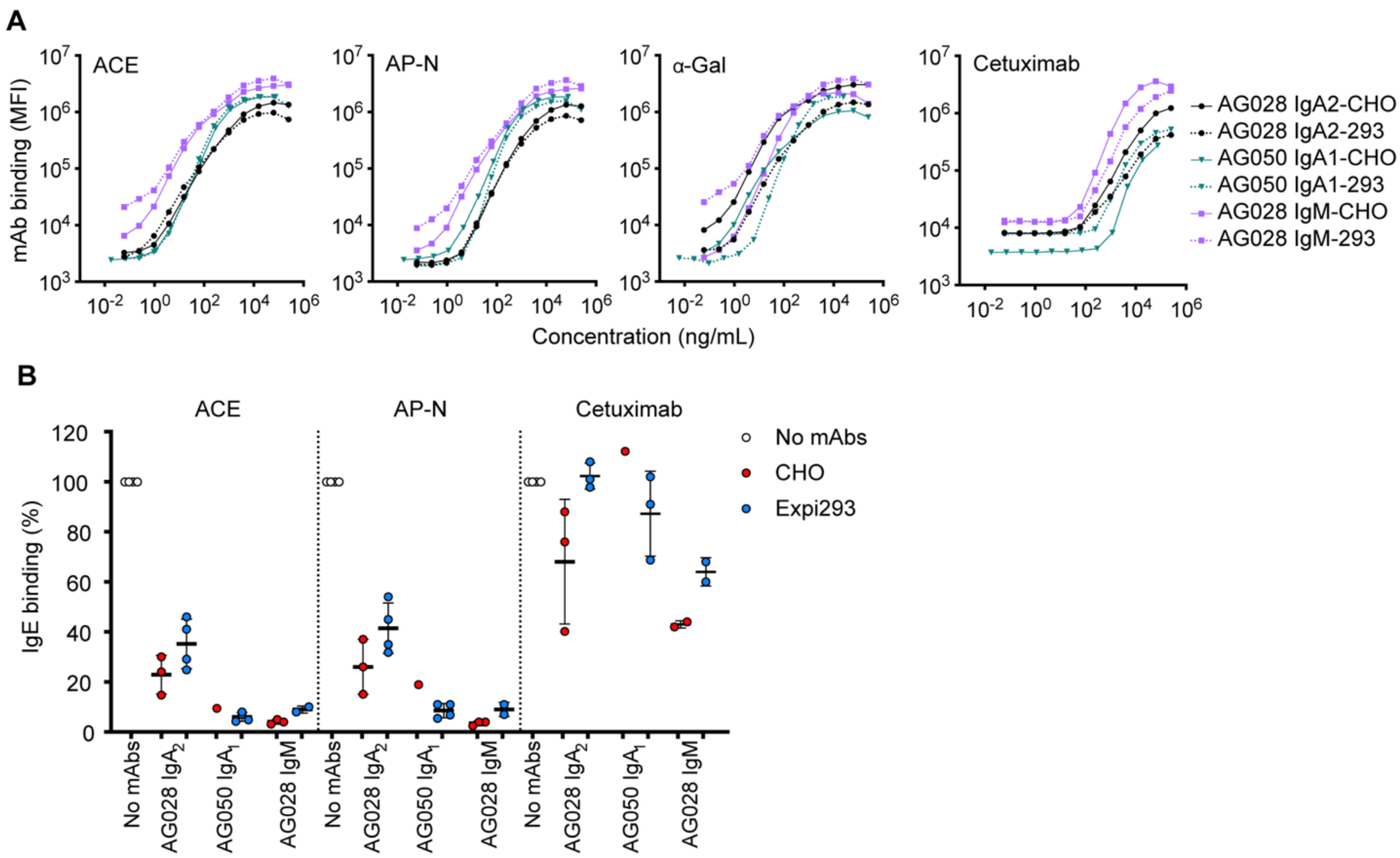

Supplemental Figure 6. Comparison of CHO cell- versus 293 cell-generated AG028 IgA_2, AG050 IgA_1, and AG028 IgM. (A) Titration curves (1:4 dilution) of mAb binding to synthetic α-Gal-conjuated to BSA, ACE, AP-N, and cetuximab. **(B)** Inhibition of polyclonal IgE binding of the UNC0209.1 plasma sample to ACE, AP-N, and cetuximab. Mean ± SD of experiments except AG050 IgA_1 generated in CHO cells.

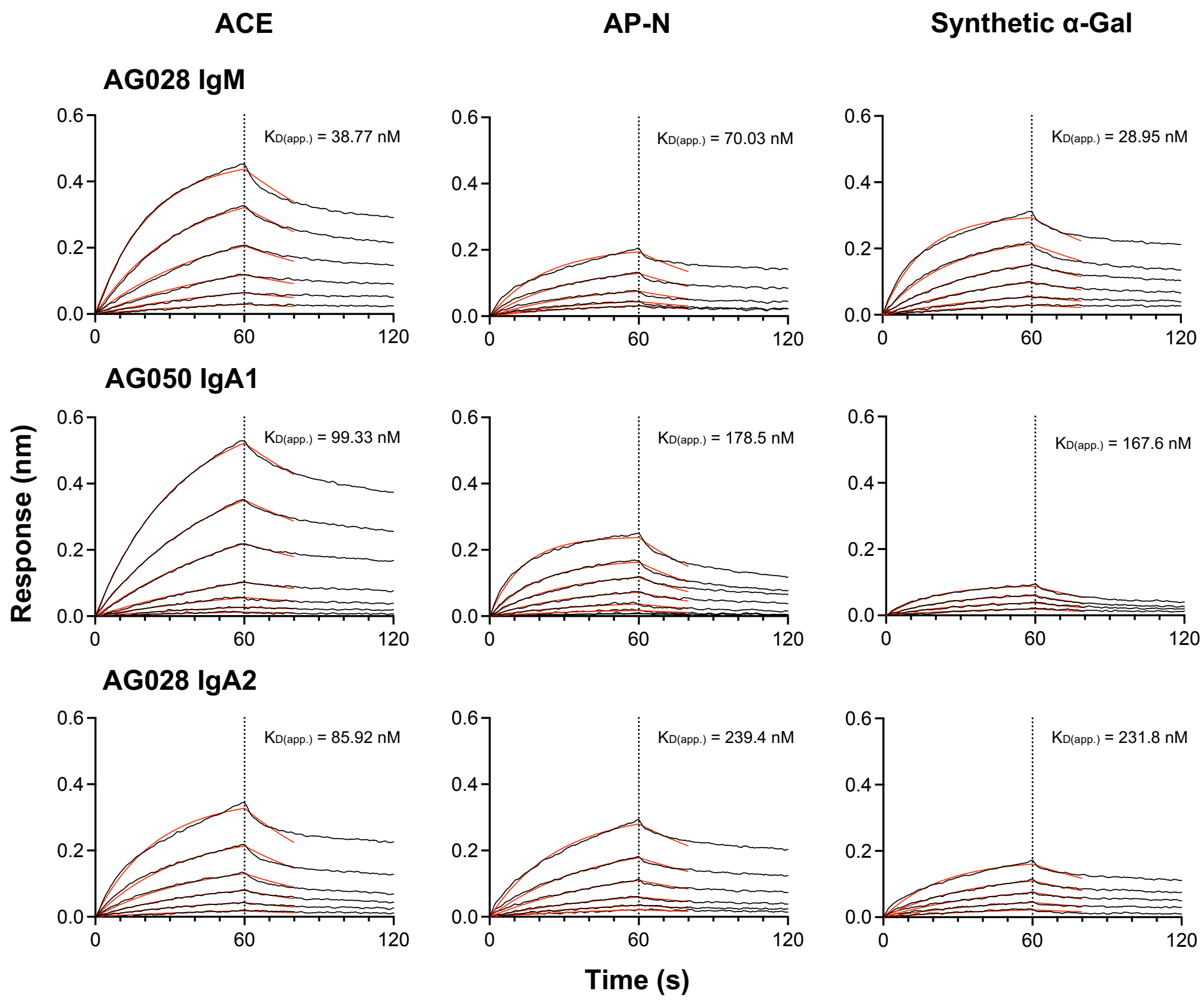

Supplemental Figure 7. Binding kinetics of AG028 IgA2, AG050 IgA1, and AG028 IgM to AGS allergens. Representative biolayer interferometry (BLI) sensorgrams (black) and corresponding fitted curves (red) show the interactions between anti-α-Gal antibodies and AGS allergens. Binding kinetics were derived from fits using at least four concentrations. The apparent affinities for each antibody are indicated within the respective sensorgrams.

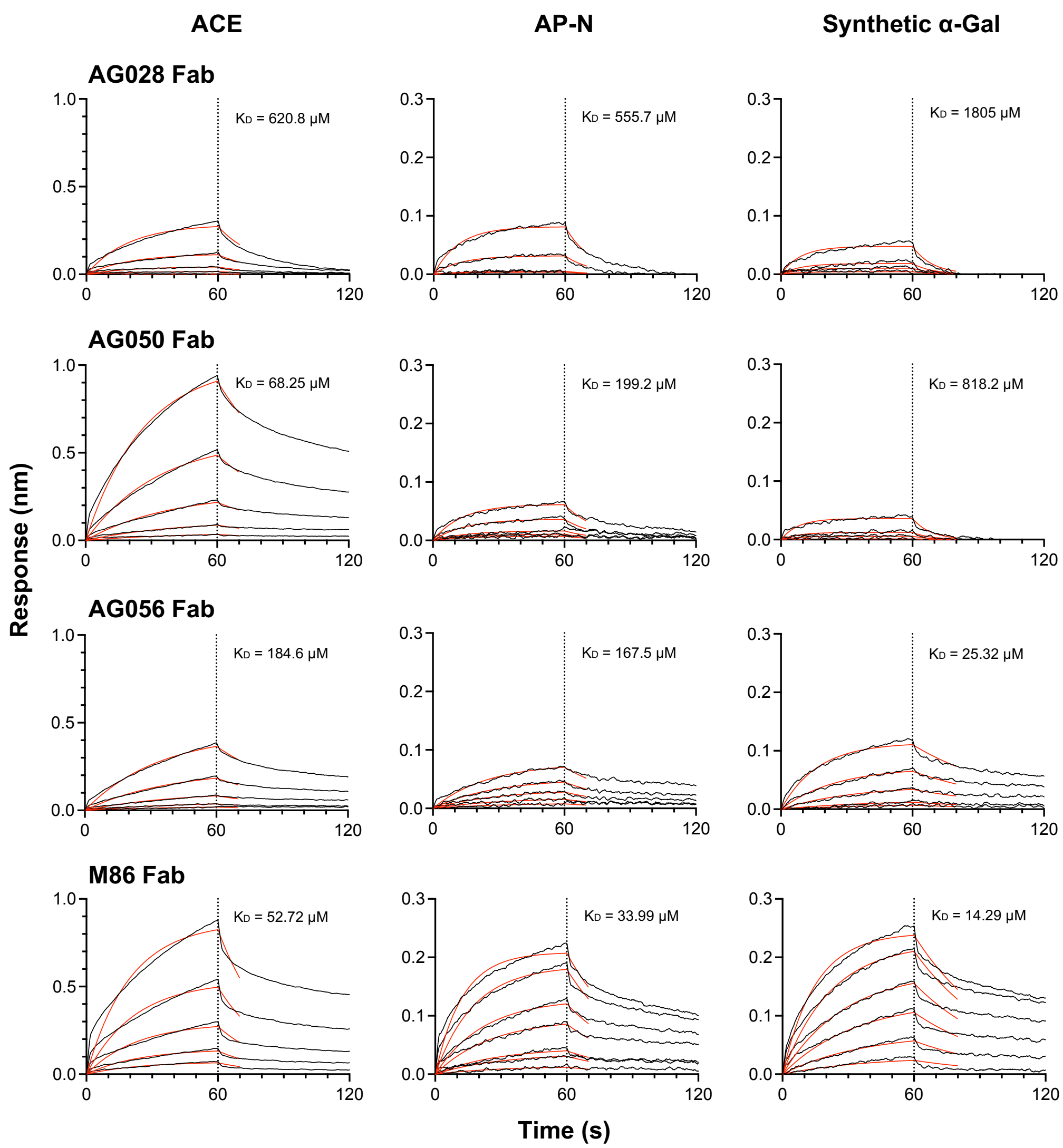

Supplemental Figure 8. Binding kinetics of AG028, AG050, AG056 and M86 Fabs to AGS allergens. Representative biolayer interferometry sensorgrams (black) and corresponding fitted curves (red) show the interactions between anti-α-Gal Fab fragments and AGS allergens. Binding kinetics were derived from fits using at least four concentrations. The calculated binding affinities for each Fab are indicated within the respective sensorgrams.

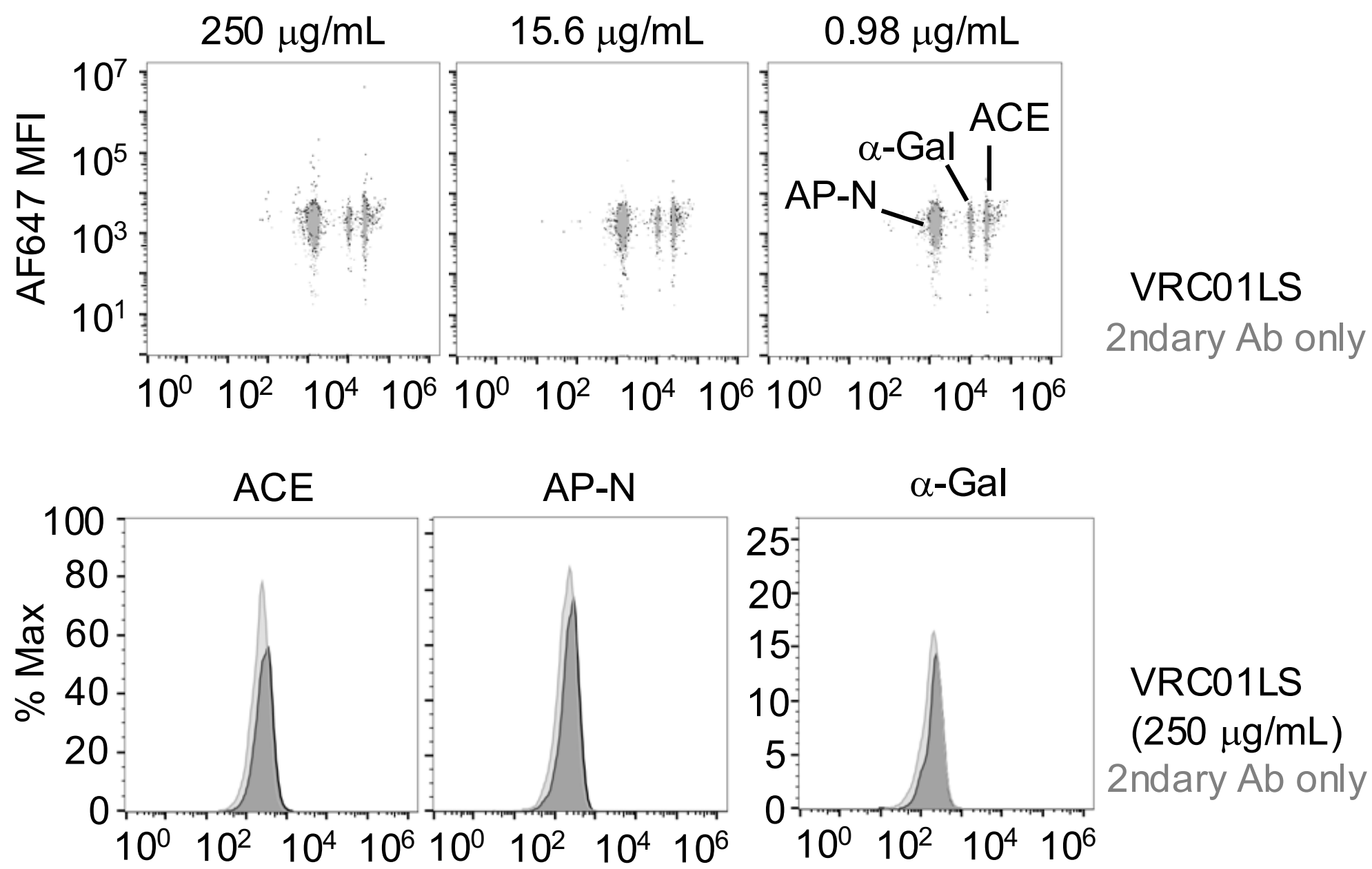

Supplemental Figure 9. VRC01LS IgG$_1$ does not bind to ACE, AP-N or synthetic α-Gal-conjugated to BSA. (A) 1:8 titration of VRC01LS IgG$_1$ (250-0.98 μg/mL) with beads conjugated to ACE, AP-N, and synthetic α-Gal. **(B)** Histograms of two overlay flow cytometry plots: VRC01LS (black) and secondary Ab control only (grey).

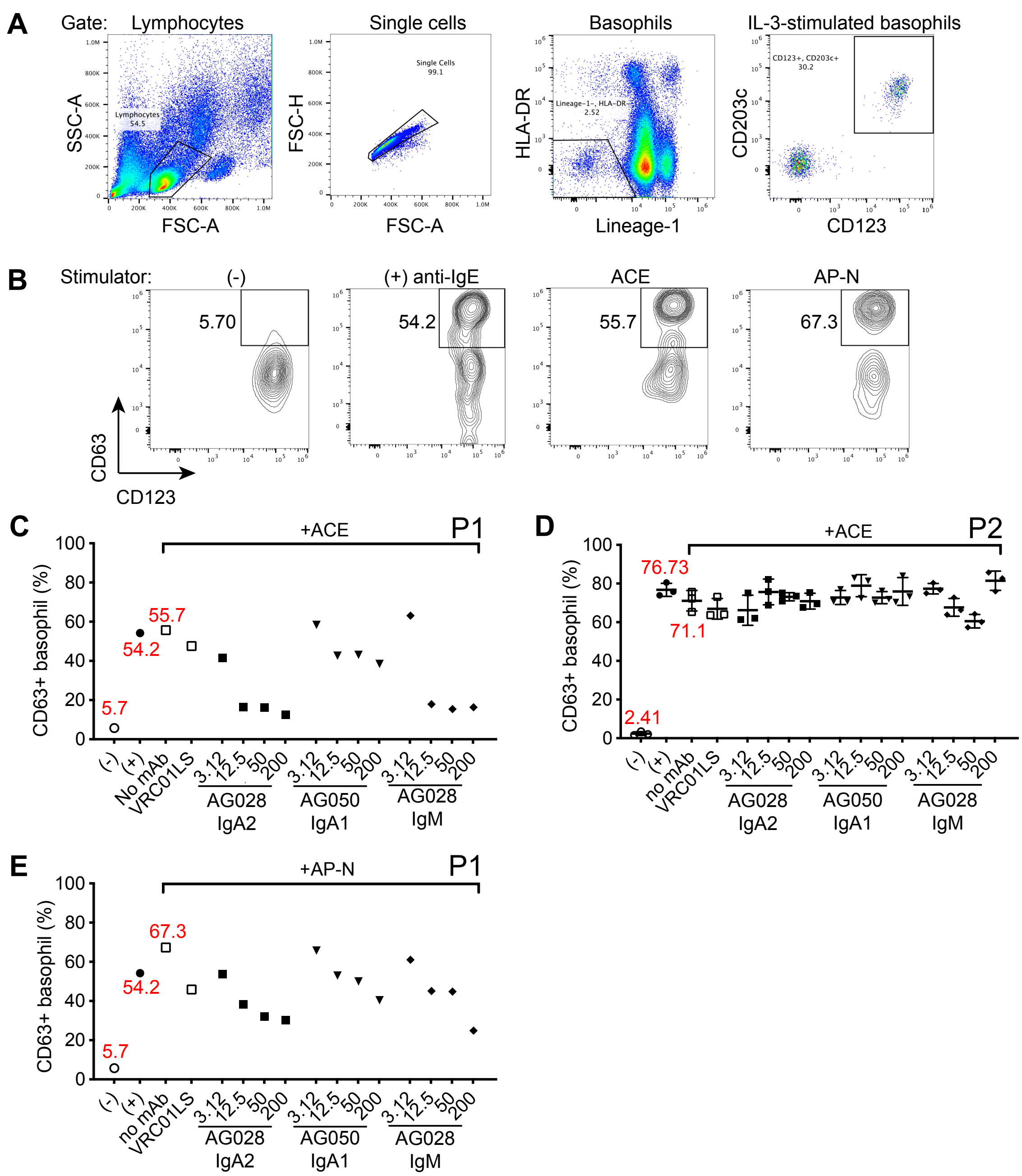

Supplementary Figure 10. Flow cytometry gating strategy for the indirect basophil activation test (BAT). Basophils within PBMCs from healthy donors were stripped of surface IgE using cold lactic acid and sensitized overnight with serum from two patients with AGS (P1 and P2). Sensitized cells were then stimulated for 30 min at 37°C with IL-3 (2 ng/mL) and ACE or AP-N (1 µg/mL), in the absence or presence of either control or anti-α-Gal mAbs (3.12, 12.5, 50, or 200 µg/mL). **(A)** Representative flow cytometry plots showing the gating strategy for CD63^{+}

basophils in P1 serum-sensitized cells. Lymphocytes were first gated by forward and side scatter, followed by singlet gating. Basophils were then identified by exclusion of HLA-DR and Lineage-1 positive cells, and IL-3-stimulated basophils were defined as $CD123^{+}$ $CD203c^{+}$ cells. **(B)** Comparison of basophil activation (as detected by increased CD63 expression) under each stimulation condition. (−) indicates unstimulated basophils, and (+) indicates basophils stimulated with anti-human IgE antibody as a positive control. ACE and AP-N were used as stimulators. **(C)** Basophils sensitized with P1 serum and stimulated with ACE. **(D)** Basophils sensitized with P2 serum and stimulated with ACE. **(E)** Basophils sensitized with P1 serum and stimulated with AP-N. In panels **(C–E)**, (−) indicates no stimulation, (+) indicates stimulation with anti-human IgE as a positive control, and VRC01LS was used as a negative mAb control. P2 samples were analyzed in triplicate. Due to limited availability of P1 serum, P1 samples were analyzed once. Percentages of $CD63^{+}$ basophils are shown in red. In **(D)**, the value shown represents the mean of triplicate measurements of $CD63^{+}$ basophils.

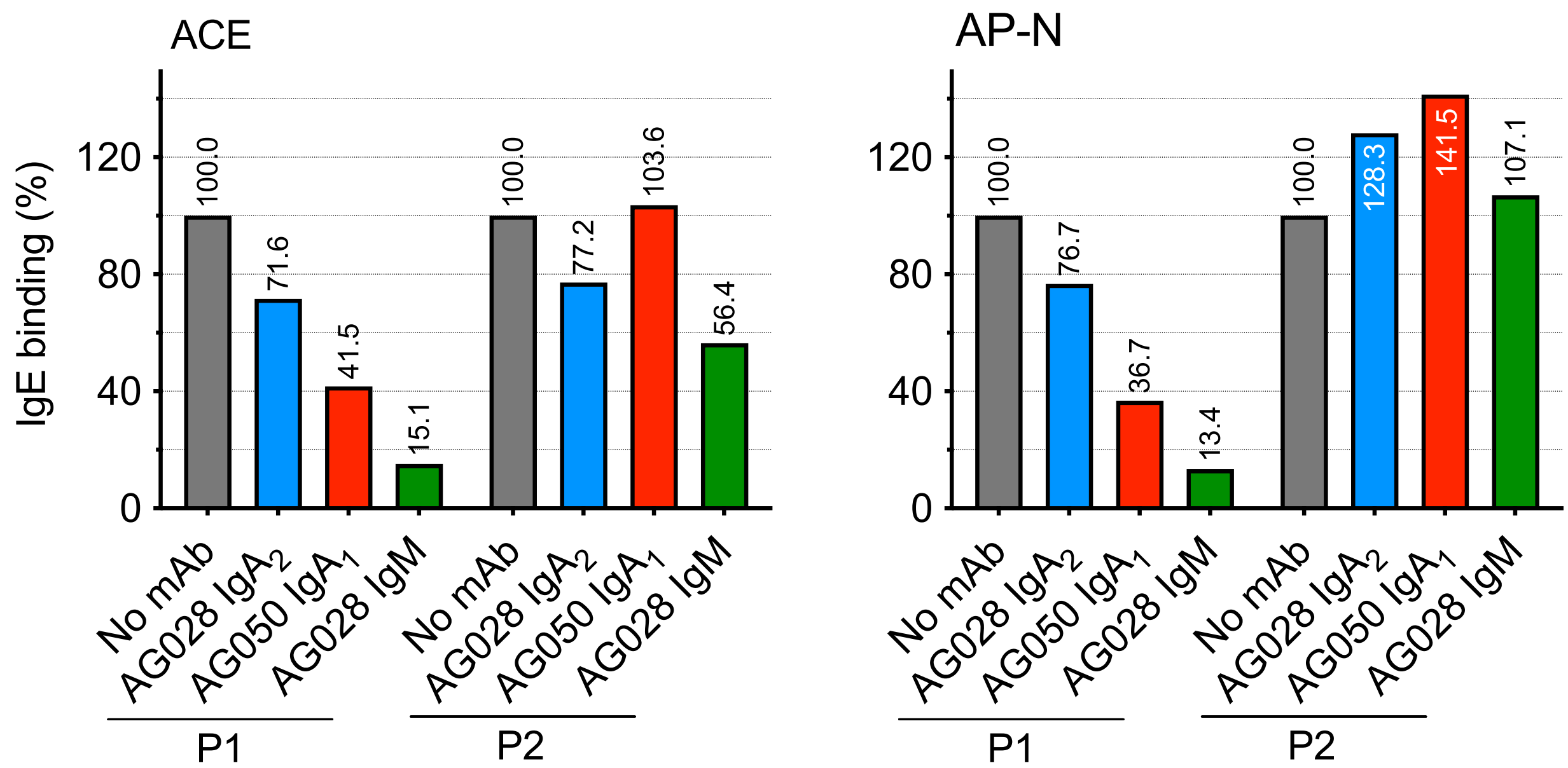

Supplemental Figure 11. Polyclonal IgE binding of AGS patient serum to ACE and AP-N in the presence of anti-α-Gal mAbs. The impact of AG028 IgA_2, AG050 IgA_1, and AG028 IgM mAbs (at a concentration of 50 μg/mL) was assessed on the polyclonal IgE binding of diluted AGS plasma samples (P1: UNC0445.1 and P2: UNC0512.1). Values indicate the percentage of serum IgE binding relative to the no mAb control.

Supplemental Table 1. Selected Malian PBMC donors for α-Gal-specific mAb isolation

ID	Age (year)	Date of Sample Collection	Sex	Prior *Plasmodium* Exposure
204	15	October 2017	F	YES
219	15	October 2017	M	YES
275	14	October 2017	F	YES
367	14	October 2017	F	YES
405	13	October 2017	F	YES
447	13	October 2017	M	YES
506	12	May 2017	M	YES
673	6	October 2017	F	YES
692	8	October 2017	F	YES
817	37	October 2017	F	YES
819	39	October 2017	F	YES
825	1	October 2017	F	YES
847	2	October 2017	M	YES
854	2	October 2017	F	YES
884	2	October 2017	M	YES
890	2	October 2017	F	YES
1016	32	October 2017	F	YES
1020	25	May 2017	M	YES

Prior malaria exposure was confirmed by microscopic examination of thick blood smears for the presence of *P. falciparum*.

Supplemental Table 2. Anti-α-gal immunoglobulin V-gene family usage

mAb	Donor ID	VH Isotype	VH	Kabat position 33 in CDRH1	VH mutation frequency (nt, %)	DH	JH	CDRH3 length (aa)	VL Isotype	VL	VL mutation frequency (nt, %)	JL
AG004	506	IgG3	3-72*01	Y	9.52	6-13*01	4*02	10	κ	2-24*01	6.46	2*01
AG008	1020	IgG3	3-30-3*01	A	7.64	3-22*01	4*02	17	κ	1-5*03	5.38	1*01
AG013	825	IgM	3-7*03	W	4.86	4-23*01	2*01	10	λ	2-8*01	1.04	1*01
AG015	825	IgM	3-7*03	W	3.82	3-10*01	4*02	11	κ	3D-20*01	3.55	1*01
AG017	825	IgM	3-9*01	A	4.17	2-2*02	4*02	15	κ	1-39*01	6.45	4*01
AG018	825	IgM	3-7*03	W	6.94	6-19*01	4*02	10	λ	7-46*01	2.43	3*02
AG020	817	IgM	3-23*01	A	5.90	2-2*01	6*04	15	κ	3-20*01	3.90	1*01
AG027	447	IgA1	3-7*01	W	11.11	3-10*01	4*01	11	κ	2-30*02	4.42	2*01
AG028	819	IgA2	6-1*01	S	12.12	4-11*01	6*03	17	λ	2-11*01	4.51	2*01
AG029	819	IgA2	3-23*01	T	11.46	1-26*01	4*02	13	κ	3-11*01	5.73	2*03
AG030	819	IgM	3-23*01	A	3.82	2-2*01	6*03	20	κ	4-1*01	1.35	5*01
AG032	204	IgM	3-48*03	E	4.17	5-18*01	4*02	6	κ	1-39*01	7.89	1*01
AG037	405	IgM	3-7*01	W	10.42	3-3*01	5*02	11	κ	2-29*03	6.12	1*01
AG039	275	IgG2	3-23*01	A	6.25	3-22*01	3*02	18	κ	3-15*01	3.94	1*01
AG041	692	IgG1	3-15*01	W	8.16	4-11*01	5*02	9	κ	1-5*03	3.94	1*01
AG045	367	IgA1	3-72*01	F	5.78	6-6*01	4*02	10	κ	2D-24*01	5.44	4*01
AG047	367	IgA1	3-66*02	D	7.72	2-21*01	4*02	7	κ	3-11*01	1.79	1*01
AG049	673	IgA2	3-74*01	W	7.29	1-26*01	3*02	13	λ	7-43*01	2.78	3*02
AG050	673	IgA1	3-30*02	G	5.90	1-1*01	6*02	15	λ	2-14*03	4.17	2*01
AG051	367	IgM	3-7*01	W	5.90	5-12*01	4*02	13	κ	3-11*01	5.38	1*01
AG053	673	IgM	3-7*03	W	7.64	5-24*01	4*02	11	λ	2-8*01	4.17	3*02
AG056	673	IgM	1-46*01	Y	6.32	5-12*01	4*02	13	λ	7-43*01	1.85	1*01
AG057	673	IgM	3-23*04	A	11.46	2-2*01	5*02	20	κ	4-1*01	2.08	3*01
AG058	673	IgM	3-23*03	A	2.08	2-8*01	4*02	21	κ	4-1*01	0.67	2*01
AG060	673	IgM	3-7*03	W	3.47	3-3*01	4*02	11	κ	2-30*02	2.02	2*01
AG061	673	IgM	3-7*03	W	7.99	3-3*01	4*02	11	κ	2-30*02	2.72	2*01
AG062	673	IgM	7-4-1*02	A	8.68	2-15*01	4*02	16	κ	1-5*03	2.38	2*01
AG067	673	IgM	4-4*07	S	2.43	5-18*01	4*02	13	κ	2-30*02	2.87	1*01
AG068	673	IgM	3-7*03	W	5.61	6-19*01	4*02	11	κ	2-30*02	1.02	2*01
AG069	673	IgM	3-49*04	A	3.47	5-18*01	3*01	12	κ	3-11*01	3.06	1*01
AG072	673	IgM	3-11*01	Y	3.74	1-26*01	4*02	15	κ	1-33*01	7.89	3*01
AG073	673	IgM	3-23*04	A	0.00	2-2*01	4*02	15	κ	3-20*01	0.00	1*01
AG074	673	IgM	3-48*04	H	5.21	1-20*01	6*02	12	λ	2-8*01	3.55	2*01
AG076	854	IgM	3-7*01	T	8.68	5-24*01	5*01	11	λ	2-8*01	1.74	1*01
AG081	890	IgM	1-3*01	T	5.56	3-22*01	4*02	15	κ	4-1*01	3.47	2*01
AG082	890	IgM	3-7*01	W	4.51	6-13*01	4*02	16	κ	3D-7*01	2.12	4*01
AG084	890	IgM	1-2*06	F	5.21	4-23*01	3*02	14	λ	7-46*01	4.96	2*01
AG085	890	IgM	1-2*06	F	5.21	4-17*01	2*01	17	κ	4-1*01	1.39	1*01
AG086	890	IgM	3-23*01	A	6.25	2-21*01	4*02	15	λ	3-20*01	3.70	2*01
AG087	890	IgM	3-7*01	W	6.25	ND	6*02	7	λ	10-54*01	4.96	3*02
AG088	890	IgM	3-15*01	W	3.40	6-13*01	5*02	14	κ	1-17*03	1.79	2*03
AG089	890	IgM	3-23*01	A	7.99	4-23*01	1*01	10	κ	1-27*01	4.30	1*01

V, variable region; H, heavy chain; L, light chain; κ, Kappa; λ, Lambda; nt, nucleotides; aa, amino acid.

Supplemental Table 3. Clinical features and serum α-Gal-specific IgE levels of AGS patients

ID	Age	Sex	Date of Sample collection	α-Gal sIgE (IU)	Total IgE (IU)	Reactions including anaphylaxis	Dairy tolerance	GI symptoms only	Bites or stings	Fume reactive
UNC0023.1	39	F	12.20.2016	2.8	431	Y	N	N	Y	Y
UNC0042.2	56	F	06.15.2022	0.17	27.4	Y	N	N	N	Y
UNC0055.1	42	F	04.19.2017	5.29	84.5	Y	N	Y*	Y	Y
UNC0061.1	74	M	04.26.2017	11.1	505	Y	Y	N	Y	N
UNC0064.1	49	F	05.10.2017	16.3	141	Y	Y	N	Y	N
UNC0064.7	50	F	01.08.2018	4.22	214	Y	Y	N	Y	N
UNC0083.1	38	F	06.21.2017	29.6	182	Y	N	N	Y	Y
UNC0084.1	68	M	06.29.2017	34.1	184	N	Y	N	Y	N
UNC0093.1	72	M	07.19.2017	82.6	210	Y	Y	N	Y	N
UNC0093.4	73	M	09.19.2018	21.5	133	Y	Y	N	Y	N
UNC0104.1	52	M	08.16.2017	15.3	237	N	Y	N	Y	N
UNC0108.1	37	M	09.28.2017	6.41	84.4	Y	N	N	Y	N
UNC0113.1	50	F	10.18.2017	6.7	78	N	Y	N	Y	N
UNC0114.1	30	F	10.18.2017	4.15	45.3	Y	N	Y*	Y	Y
UNC0116.1	48	F	10.25.2017	1.84	51.9	Y	N	N	Y	N
UNC0118.1	45	F	11.29.2017	28.2	136	Y	Y	N	Y	N
UNC0119.1	35	F	12.05.2017	1.21	383	N	N	Y	N	N
UNC0120.1	43	M	12.05.2017	1.34	42.2	N	Y	N	Y	N
UNC0121.1	74	M	12.06.2017	40	303	N	Y	N	Y	N
UNC0131.1	41	F	03.21.2018	6.75	21	Y	N	N	Y	N
UNC0137.1	54	M	04.11.2018	6.23	301	Y	N	N	Y	N
UNC0138.1	69	M	05.08.2018	1.57	144	Y	Y	Y*	Y	N
UNC0146.2	45	F	08.20.2019	88.6	391	Y	N	N	Y	N
UNC0147.1	65	M	07.11.2018	>100	274	Y	Y	N	Y	N
UNC0150.1	53	M	09.25.2018	5.77	185	Y	N	N	Y	N
UNC0209.1	70	M	12.09.2020	68	815	N	N	Y	Y	N
UNC0209.2	71	M	04.20.2021	27.8	484	N	N	Y	Y	N
UNC0220.1	28	M	02.17.2021	>100	1029	Y	N	N	Y	Y
UNC0228.1	71	F	04.14.2021	2.76	277	Y	N	N	Y	N
UNC0238.1	67	M	05.25.2021	0.1	277	Y	Y	N	Y	N
UNC0241.1	68	M	06.02.2021	37.22	339	Y	N	N	Y	N
UNC0243.1	59	M	06.09.2021	1.96	27.6	N	Y	N	Y	N
UNC0244.1	56	M	06.09.2021	0.5	5.42	N	Y	N	Y	N
UNC0245.1	74	M	06.15.2021	2.24	176	N	Y	N	Y	N
UNC0246.1	77	F	06.16.2021	1.02	46.6	Y	N	N	Y	N
UNC0247.1	48	M	06.16.2021	0.18	180	N	Y	N	Y	N
UNC0248.1	75	M	06.16.2021	1.67	37	Y	N	N	Y	N
UNC0249.1	51	F	06.16.2021	0.42	62	N	Y	N	Y	N
UNC0250.1	53	F	06.23.2021	0.32	52.6	N	Y	N	Y	N
UNC0251.1	58	F	06.23.2021	>100	433	Y	N	N	Y	N
UNC0252.1	73	M	06.23.2021	60.1	49.4	N	Y	N	Y	N
UNC0264.1	54	F	07.28.2021	9.6	33.6	Y	N	N	Y	N
UNC0265.1	65	F	07.28.2021	2.21	448	Y	N	Y*	Y	Y
UNC0268.1	43	F	08.25.2021	7.38	134	Y	N	N	Y	Y
UNC0271.1	56	F	09.01.2021	3.25	21.6	Y	Y	N	Y	N
UNC0272.1	73	M	09.08.2021	2.2	105	N	Y	N	Y	N
UNC0273.1	61	F	09.08.2021	4.09	13.1	N	Y	N	Y	N
UNC0274.1	71	F	09.08.2021	1.17	34.4	N	Y	Y	Y	N
UNC0275	52	F	09.08.2021	77.6	449	N	Y	Y	Y	N
UNC0289.1	75	M	09.15.2021	25.5	132	N	Y	N	Y	N
UNC0294.1	63	F	09.29.2021	0.16	115	Y	N	Y*	Y	N
UNC0303.1	46	F	10.20.2021	4.32	138	N	Y	Y	Y	N
UNC0305.1	66	M	10.27.2021	11	68.1	N	Y	N	Y	N
UNC0306.1	73	M	10.27.2021	17.7	180	Y	Y	N	Y	N
UNC0311.1	55	F	11.03.2021	1.05	5.58	N	N	Y	Y	Y

UNC0314.1	41	M	11.17.2021	15.7	673	N	Y	N	Y	N
UNC0323.1	60	M	12.08.2021	38.4	212	N	Y	N	Y	N
UNC0327.1	45	F	01.19.2022	0.75	27.5	Y	N	Y[A]	Y	N
UNC0328.1	42	F	02.09.2022	2.97	60.4	N	N	Y	Y	Y
UNC0329.1	49	F	02.09.2022	1.63	13.1	N	Y	N	Y	N
UNC0330.1	72	F	02.09.2022	31.5	184	Y	Y	N	Y	N
UNC0332.1	49	F	02.16.2022	0.14	144	Y	N	N	Y	N
UNC0334.1	77	F	02.23.2022	7.6	22.9	Y	N	N	Y	N
UNC0335.1	23	M	02.23.2022	11	140	Y	Y	N	N	Y
UNC0336.1	76	F	02.23.2022	0.8	61.1	N	N	Y	N	N
UNC0341.1	70	M	03.09.2022	8.77	1223	Y	Y	N	Y	N
UNC0343.1	72	F	03.23.2022	0.62	839	N	Y	N	Y	N
UNC0344.1	23	F	03.23.2022	5.24	253	Y	N	N	Y	Y
UNC0348.1	77	M	04.06.2022	11	160	N	Y	N	Y	N
UNC0350.1	61	F	04.13.2022	7.26	78.4	N	Y	N	Y	N
UNC0352.1	55	M	04.20.2022	3.86	2348	N	Y	Y	Y	N
UNC0353.1	52	F	04.20.2022	14.9	85.7	Y	Y	N	Y	N
UNC0355.1	61	F	04.27.2022	18.4	56.8	N	Y	N	Y	N
UNC0356.1	62	F	05.04.2022	2.9	34.6	Y	N	N	Y	Y
UNC0357.1	63	F	05.04.2022	>100	848	Y	Y	N	Y	N
UNC0358.1	68	M	05.04.2022	2.43	57.2	N	Y	N	Y	N
UNC0360.1	30	F	05.11.2022	0.1	210	N	N	Y	Y	Y
UNC0361.1	50	F	05.11.2022	6.93	45.8	N	Y	N	Y	N
UNC0362.1	63	F	05.11.2022	4.16	698	Y	N	N	Y	Y
UNC0364.1	37	F	06.01.2022	22.3	432	Y	N	N	Y	Y
UNC0367.1	57	F	06.08.2022	2.5	45.7	Y	N	Y*	Y	Y
UNC0368.1	77	F	06.15.2022	0.46	58.4	N	N	Y	Y	Y
UNC0380.1	41	M	07.20.2022	36.5	663	N	Y	Y	Y	N
UNC0383.1	74	F	07.27.2022	>100	757	Y	N	N	Y	Y
UNC0384.1	20	F	07.27.2022	60.4	408	Y	N	N	Y	Y
UNC0391.1	65	M	09.07.2022	>100	584	Y	Y	N	Y	N
UNC0445.1	56	F	05.03.2023	24.1	156	N	N	Y	Y	N
UNC0512.1	71	M	03.13.2024	70.7	686	Y	Y	N	Y	N

[A]On occasion

Supplemental Table 4. Summary of binding association (k_{on}), dissociation (k_{dis}), and K_D of anti-α-Gal antibodies.

Allergen	Antibody	K_D (M)	K_D Error	k_{on} (1/Ms)	k_{on} Error	k_{off} (1/s)	k_{off} Error
ACE	AG028 IgA2	8.592E-08	1.809E-09	2.214E05	4.225E03	1.903E-02	1.694E-04
	AG050 IgA1	9.933E-08	8.132E-10	1.012E05	6.427E02	1.006E-02	5.200E-05
	AG028 IgM	3.877E-08	4.740E-10	3.311E05	2.983E03	1.284E-02	1.061E-04
	AG028 Fab	6.208E-04	2.293E-04	7.834E01	2.889E01	4.863E-02	9.849E-04
		6.996E-04	3.039E-04	7.972E01	3.459E01	5.577E-02	1.180E-03
	AG050 Fab	6.825E-05	2.755E-06	3.296E02	1.208E01	2.250E-02	3.814E-04
		8.279E-05	3.395E-06	2.805E02	1.074E01	2.322E-02	3.415E-04
	AG056 Fab	1.846E-04	1.980E-05	1.269E02	1.340E01	2.343E-02	4.409E-04
		1.192E-04	8.397E-06	1.988E02	1.350E01	2.370E-02	4.385E-04
	M86 Fab	5.272E-05	6.933E-06	7.822E02	1.018E02	4.124E-02	7.773E-04
		7.091E-05	1.475E-05	6.290E02	1.300E02	4.460E-02	1.024E-03
AP-N	AG028 IgA2	2.394E-07	4.455E-09	5.668E04	8.752E02	1.357E-02	1.409E-04
	AG050 IgA1	1.785E-07	2.009E-09	1.287E05	1.196E03	2.296E-02	1.460E-04
	AG028 IgM	7.003E-08	2.167E-09	2.638E05	7.138E03	1.848E-02	2.775E-04
	AG028 Fab	5.557E-04	1.902E-04	1.459E02	4.985E01	8.109E-02	1.663E-03
	AG050 Fab	1.992E-04	2.822E-05	3.079E02	4.314E01	6.132E-02	1.283E-03
	AG056 Fab	1.675E-04	1.990E-05	1.889E02	2.210E01	3.164E-02	6.572E-04
	M86 Fab	3.40E-05	9.84E-07	9.89E+02	2.44E+01	3.36E-02	5.11E-04
		5.458E-05	2.057E-06	7.571E02	2.622E01	4.132E-02	6.139E-04
Synthetic α-Gal	AG028 IgA2	2.318E-07	5.679E-09	6.722E04	1.381E03	1.558E-02	2.080E-04
	AG050 IgA1	1.676E-07	4.167E-09	1.450E05	3.281E03	2.430E-02	2.501E-04
	AG028 IgM	2.895E-08	4.057E-10	4.761E05	4.568E03	1.378E-02	1.408E-04
	AG028 Fab	1.846E-04	1.980E-05	1.269E02	1.340E01	2.343E-02	4.409E-04
	AG050 Fab	8.182E-04	5.899E-04	1.498E02	1.079E02	1.226E-01	2.935E-03
	AG056 Fab	2.532E-05	6.881E-07	8.169E02	1.810E01	2.068E-02	3.256E-04
	M86 Fab	1.429E-05	3.146E-07	1.747E03	3.445E01	2.497E-02	2.447E-04

Supplemental Reference

1. Tan J, et al. Functional human IgA targets a conserved site on malaria sporozoites. *Sci Transl Med.* 2021;13(599).
